# Electrochemical Impedance Spectroscopy of All-Perovskite Tandem Solar Cells

**DOI:** 10.1021/acsenergylett.3c02018

**Published:** 2024-01-11

**Authors:** Bart Roose, Krishanu Dey, Melissa R Fitzsimmons, Yu-Hsien Chiang, Petra J Cameron, Samuel D Stranks

**Affiliations:** † Department of Chemical Engineering and Biotechnology, University of Cambridge, Philippa Fawcett Drive, Cambridge CB3 0AS, U.K.; ‡ Department of Physics, Cavendish Laboratory, University of Cambridge, 19 JJ Thomson Avenue, Cambridge CB3 0HE, U.K.; § Department of Chemistry, University of Bath, Claverton Down, Bath BA2 7AY, U.K.

## Abstract

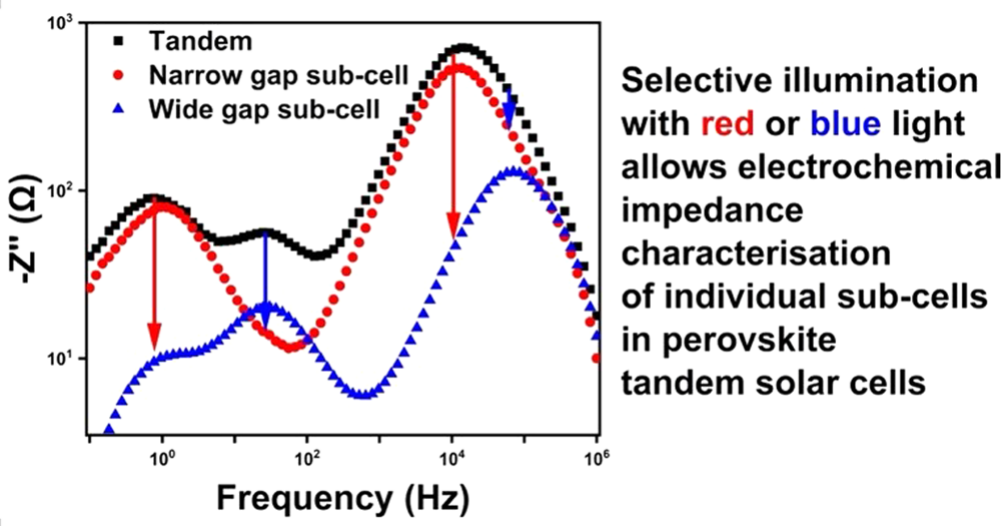

This work explores electrochemical impedance spectroscopy to study recombination and ionic processes in all-perovskite tandem solar cells. We exploit selective excitation of each subcell to enhance or suppress the impedance signal from each subcell, allowing study of individual tandem subcells. We use this selective excitation methodology to show that the recombination resistance and ionic time constants of the wide gap subcell are increased with passivation. Furthermore, we investigate subcell-dependent degradation during maximum power point tracking and find an increase in recombination resistance and a decrease in capacitance for both subcells. Complementary optical and external quantum efficiency measurements indicate that the main driver for performance loss is the reduced capacity of the recombination layer to facilitate recombination due to the formation of a charge extraction barrier. This methodology highlights electrochemical impedance spectroscopy as a powerful tool to provide critical feedback to unlock the full potential of perovskite tandem solar cells.

Perovskite solar cells (PSCs) have made great progress since their first report in 2009.^1^ Record lab-scale power conversion efficiency (PCE) is practically on-par with established silicon technology,^2^ and further improvements are still expected.^3^ This rapid development can be attributed to the excellent optoelectronic properties of halide perovskites and their compatibility with inexpensive processing methods.^4,5^ PSCs show potential to be fabricated cost-effectively using high-throughput methods^6^ in geographically distributed facilities.^7^

The bandgap of metal halide perovskites can be tuned by altering the chemical composition, making these materials strong candidates for tandem solar cells.^8−13^ A tandem solar cell consists of two subcells stacked on top of each other. Each subcell absorbs a different region of the solar spectrum, which allows tandem solar cells to harvest a broader range of wavelengths more efficiently than a single absorber alone. In all-perovskite tandem solar cells, the narrow bandgap subcell typically uses an alloyed lead/tin pure iodide perovskite (APb_*x*_Sn_1–*x*_I_3_), with a bandgap of ∼1.2–1.25 eV. The wide bandgap subcell uses an APb(I_*x*_Br_1–*x*_) perovskite with bromide fractions up to 40% and a bandgap of ∼1.7–1.8 eV.^14^ The radiative efficiency limit of a tandem solar cell is ∼45%, compared to ∼33% for a single junction (SJ) solar cell.^15^ Tandem solar cells can thus significantly reduce the $/W cost of photovoltaics and further speed up the renewable energy transition.^16^ Remarkable progress in all-perovskite tandem efficiency has been achieved by improving the wide bandgap subcell,^17,18^ the narrow bandgap subcell,^14,19,20^ and the recombination junction.^21^ However, all-perovskite tandems have only recently surpassed SJ PSCs in PCE (26.0% for SJ versus 29.1% for all-perovskite tandem solar cells)^22^ and are still far from practical efficiency limits.^15^ A better understanding of the device properties is essential to further increase performance in a systematic, consistent, and rational fashion. However, tandem solar cells bring new challenges through the increased complexity of these devices compared to SJ cells,^23^ and new approaches need to be developed to study individual subcells or interfaces within the tandem stack.^18^

Electrochemical impedance spectroscopy (EIS) is a powerful characterization technique that can provide insights into electrical and electrochemical processes that occur during device operation. Typically, a 5–10 mV sinusoidally oscillating voltage is applied in addition to a DC voltage bias. The resulting sinusoidally varying current output is measured as a function of the frequency of the oscillating voltage. By measuring the amplitude and phase shift of the current with respect to the applied voltage, the impedance can be calculated. The excitation signal perturbs the device, and as the time it takes the system to respond (relaxation time) is process-specific, it allows the distinction of processes occurring at different time scales. More information can be extracted from the spectrum by modeling with an equivalent circuit (EC), and the values of the resulting parameters can provide information on the underlying physical nature of these different processes.^24−28^ EIS has been used extensively in SJ perovskite solar cells to study processes such as surface recombination and charge collection efficiency,^27^ interfacial and ionic reactions,^29^ and activation energies for ionic motion.^30,31^ However, EIS has not yet been applied to perovskite tandem solar cells.

Here, electrochemical impedance spectroscopy (EIS) is explored to characterize all-perovskite tandem solar cells. We show that tandem EIS spectra acquired under standard measuring conditions (i.e., full spectrum illumination) are complex and hard to resolve for individual subcells. In order to better understand the EIS of the tandem cell, we have measured the EIS for wide gap and narrow gap SJ cells. We see the expected dependence of the spectra on light intensity, where the magnitude of the impedance signal increases with decreasing intensity at all frequencies, while simultaneously there is a shift in the frequency at which the impedance reaches a maximum.^30,32^ Making use of the different bandgaps of the narrow and wide bandgap subcells, we use modified illumination spectra to selectively excite individual subcells in the tandems. This allows us to extract subcell-selective information, as we show that the impedance response is dominated by the current-limiting subcell. Increasing the intensity of the wavelengths that are only absorbed by one subcell ensures that the current is limited by the second subcell, allowing accurate time constants to be extracted for the second subcell. We use this selective illumination approach to study the effects of passivation of the wide gap subcell on the electronic recombination and ionic time constants of an all-perovskite tandem. We find that the high-frequency recombination resistance and low-frequency ionic time constants of the wide gap subcell are increased with passivation, while those of the narrow gap remain largely unchanged. In addition, we studied the effects of operating an all-perovskite tandem at the maximum power point for 24 h, during which the tandem efficiency decreases to ∼75% of the starting efficiency. At the same time, an increase in electronic recombination time constant is found for both subcells. Complementary photoluminescence (PL), UV/vis absorption, and external quantum efficiency (EQE) measurements indicate that the recombination junction becomes less efficient under operation. We demonstrate the versatility of the selective illumination EIS method to better understand all-perovskite tandems, allowing targeted approaches to further improve the efficiency and stability.

All-perovskite tandem solar cells were fabricated building on previous work,^14,33^ with perovskite absorber layers solution-processed in device stacks consisting of ITO/[2-(9*H*-carbazol-9-yl)ethyl]phosphonic acid (2PACz)/Cs_0.25_FA_0.75_Pb(I_0.767_Br_0.233_) wide gap perovskite/propane-1,3-diammonium iodide (PDAI_2_)/C60/SnO_*x*_/Au/PEDOT:PSS/Cs_0.15_FA_0.85_Pb_0.5_Sn_0.5_I_3_ narrow gap perovskite/ethane-1,2-diammonium iodide (EDAI_2_)/C60/BCP/Cu (see inset of Figure 1a). An average power conversion efficiency (PCE) of ∼22% and a champion PCE of 23.8% (Figure 1a, Table 1) were achieved. Short circuit currents (*J*_*SC*_) match well between the *J–V* and EQE measurements (Figure S1). Performance loss of the devices is negligible on time scales (∼10 min) that are required to record an electrochemical impedance spectrum (Figure 1b).^25^ Wide gap (ITO/2PACz/wide gap perovskite/PDAI_2_/C60/BCP/Cu) and narrow gap (ITO/PEDOT:PSS/narrow gap perovskite/EDAI_2_/C60/BCP/Cu) SJ devices, analogous to the tandem subcells, were fabricated (performance statistics in Table S1) to compare EIS signals between tandems and SJs. Note that these SJ devices use identical absorber and charge transport layers as the corresponding tandem subcells.

**Figure 1:**
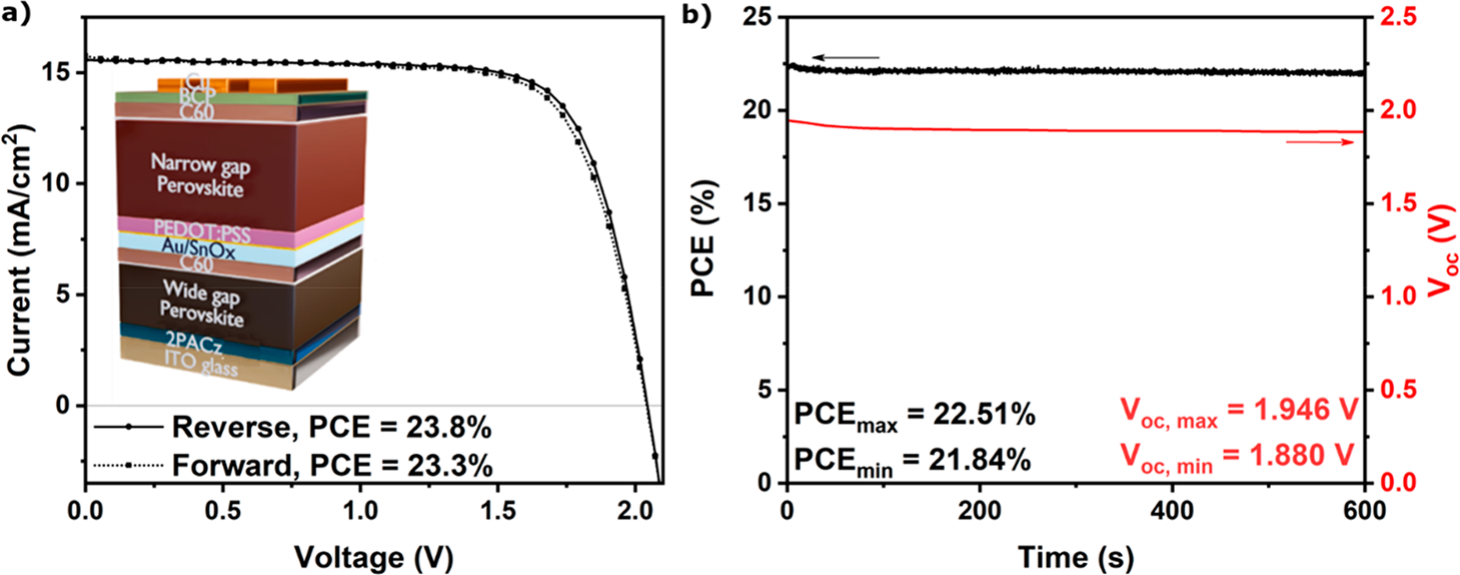
(a) *J*–*V* curve of champion efficiency all-perovskite tandem solar cell, scanned from forward to reverse bias (Reverse) and from reverse to forward bias (Forward) at a scan rate of 100 mV/s. Inset: The all-perovskite tandem device stack.^34^ (b) Maximum power point tracking (top, black line) and *V*_*OC*_ tracking (bottom, red line) of an all-perovskite tandem solar cell.

**Table 1:** Performance Parameters of All-Perovskite Tandems^a^ and the Champion Device

	*V*_*OC*_ (V)	*J*_*SC*_ (mA/cm^2^)	FF (%)	Rev PCE (%)	Fwd PCE (%)
Average	1.95 ± 0.03	15.3 ± 0.4	75.0 ± 1.5	22.3 ± 0.7	21.9 ± 0.6
Champion	2.05	15.6	74.8	23.8	23.3

a Average of 28 devices.

EIS measurements were performed at open-circuit potential (*V*_*OC*_) under illumination, using a potentiostatic excitation applied at logarithmically distributed frequencies between 10^–1^ and 10^6^ Hz (see Methods). The *V*_*OC*_ decreases in the first minutes but stabilizes after 3 min of illumination (Figure 1b). To account for the initial drop in *V*_*OC*_, devices are held at *V*_*OC*_ for 3 min before the measurement is started. The Nyquist (Figure 2a,b) and corresponding imaginary impedance (−*Z*″) vs frequency plots (Figure 2d,e) for wide gap and narrow gap SJ perovskite devices show two clear semicircles and two distinct peaks, respectively. For PSCs, the high-frequency RC time constant is related to nonradiative recombination and the geometric capacitance of the device. Note that radiative recombination is too fast to be probed with EIS. The origin of the low-frequency time constant is still debated, but a consensus is beginning to emerge that the low-frequency response is caused by the ion-modulated recombination current.^25,35^ In this work, we limit ourselves to denominating the high-frequency semicircle as the electronic recombination time constant, relating to the recombination resistance (but note that this does not encompass radiative recombination) and geometric capacitance, and the low-frequency semicircle as the ionic RC time constant, relating to ionic modulation of the recombination (or injection) current. These denominations are used for simplicity, though the true nature of the electronic and ionic processes is complex and discussed in detail elsewhere.^26,29,30,36,37^ Characteristic relaxation time constants for these processes can be expressed in terms of resistance (*R*) and capacitance (*C*), although extreme care has to be taken when assigning physical meaning to these parameters.^25^ Relaxation processes can be modeled with a resistor-capacitor equivalent circuit (EC), from which the relaxation time constant τ can be found:
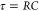


**Figure 2:**
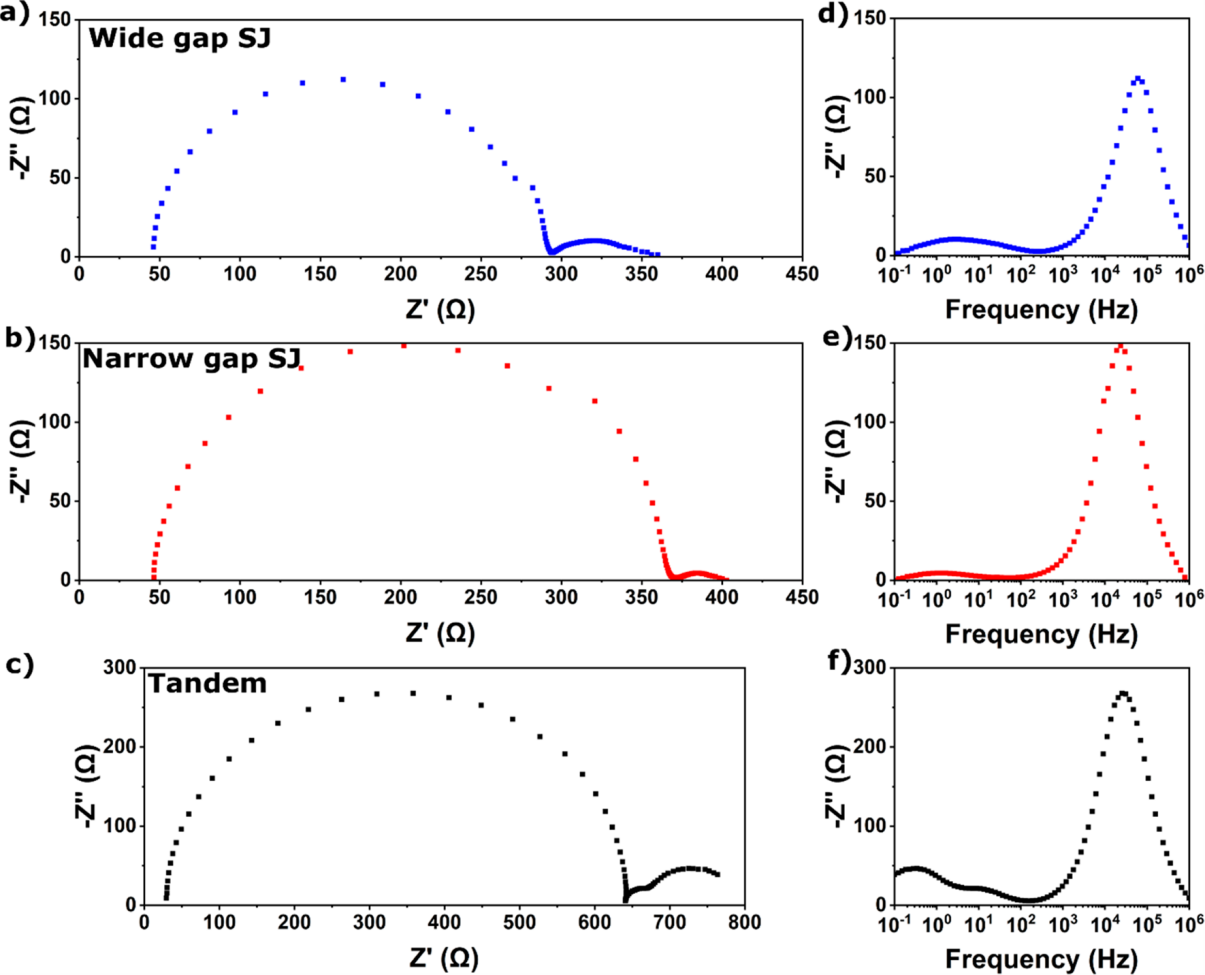
Nyquist plots of (a) wide gap SJ, (b) narrow gap SJ, and (c) all-perovskite tandem solar cells (see Tables 1 and S1 for corresponding device parameters). −*Z*″ vs frequency plots of (d) wide gap SJ, (e) narrow gap SJ, and (f) all-perovskite tandem solar cells. Devices were illuminated using full spectrum AM1.5G illumination at 0.1 sun intensity and measured at *V*_*OC*_, with a wait time of 3 min before each measurement to allow *V*_*OC*_ to stabilize. Note the different scales of the *x*-axis in (a–c).

Note, however, that for PSCs the capacitor element is often replaced by a constant phase element (CPE) to account for sample inhomogeneity.^25^ Alternatively, τ can be derived from the apex frequency (*f*) of −*Z*″:
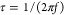


Both methods should return identical values for τ. Throughout this paper, we denote the time constant for the high-frequency arc the “electronic recombination time constant”. The time constants for all lower frequency processes are denoted “ionic time constants”, as they occur on time scales where ionic motion modulates the current measured from the devices. It is important to note that these characteristic time constants are indicative of processes occurring in the cell but do not directly give electron lifetimes. It is also important that the time constant and lifetime are not used interchangeably. Electronic recombination and ionic time constants can be readily extracted for these SJ devices by either EC fitting, using a series resistance element and two R-CPE elements in series (Figure S2a), hereafter referred to as SJ EC, or from the frequency corresponding to the apex of the Nyquist plot. Here, we will focus on the value extracted from EC fitting, as it provides a more detailed analysis, and we will use the apex frequency to verify the validity of the time constant found. Electronic recombination and ionic time constants for the devices in Figure 2 can be found in Table 2. The corresponding resistance and capacitance values are listed in Table S2. The frequency of the apex of the Nyquist plot can be displayed by plotting −*Z*″ vs frequency, allowing shifts in time constants to be elegantly visualized.

**Table 2:** Relaxation Time Constants for Electronic Recombination and Ionic Processes for the SJs and Tandem in Figure 2^a^

Device	Process	*R* (Ω)	CPE (F)	τ (s)
Wide gap SJ	Recombination	237	1.09 × 10^–8^	2.6 × 10^–6^
	Ionic	62.0	9.29 × 10^–4^	5.8 × 10^–2^
Narrow gap SJ	Recombination	320	2.12 × 10^–4^	6.8 × 10^–6^
	Ionic	24.9	5.40 × 10^–3^	1.3 × 10^–1^
Tandem	Recombination	629	9.06 × 10^–9^	5.7 × 10^–6^
	Ionic	19.4	2.79 × 10^–4^	5.4 × 10^–3^
		144	4.70 × 10^–3^	6.8 × 10^–1^

a Corresponding resistances, capacitances, and apex frequencies can be found in Table S2.

Typical Nyquist and −*Z*″ vs frequency plots for an all-perovskite tandem device are shown in Figure 2c,f, respectively. There are two distinct low-frequency semicircles, although we note that this is not always the case (Figure S3). The high-frequency signals for both subcells heavily overlap, making it difficult to extract time constants for individual processes, which is indeed impossible using the apex frequency approach. An EC consisting of a series resistance and four R-CPE elements (Figure S2b, hereafter referred to as tandem EC) can be rationalized by assuming that each subcell will have its own electronic recombination and ionic time constants. However, this approach has two major shortcomings. First, in spectra where semicircles overlap significantly, it is impossible to find a satisfactory fit for the individual relaxation time constants (HF semicircle in Figure 2c), as we have to guess where one feature ends and the next one starts. Second, even if two distinct relaxation time constants can be observed, it is not obvious which relaxation time constant belongs to which subcell, therefore making it difficult to identify performance limiting bottlenecks.

To further understand the EIS signal of the tandem, we first explored the influence of the illumination intensity on the EIS signal of the equivalent SJ devices. Wide gap and narrow gap SJ devices were illuminated using full spectrum AM1.5G light, with intensity ranging from 1.0 to 0.01 sun, while performing EIS measurements. The corresponding Nyquist and −*Z*″ vs frequency plots are displayed in Figure S4 (corresponding relaxation time constants are in Table S3). It can clearly be seen in the Nyquist plots that both *Z′* and *–Z″* increase with decreasing light intensity. An intrinsic factor contributing to this increase is that the rate of carrier generation decreases with decreasing light intensity (Figure S5). As the measurement is performed at open circuit, this means the rate of recombination also decreases. The recombination current is inversely proportional to the recombination resistance; hence, as the current decreases, the recombination resistance increases, and the diameter of the high-frequency arc in the Nyquist plot increases.^25^ Further to this, carrier lifetime generally increases with decreasing light intensity,^38^ resulting in increased impedance. Additionally, the apex frequencies of all semicircles show distinct shifts (Figure S4, Table S3).

The illumination intensity can thus be used to manipulate the magnitude and characteristic frequency of the EIS signal. Each subcell in the tandem device absorbs a range of wavelengths which the other subcell does not absorb (Figure S1). This allows us to magnify the signal from one of the subcells, while suppressing the signal from the other subcell, and allow subcell-selective measurements. The Nyquist and −*Z*″ vs frequency plots at 0.1 sun full spectrum AM1.5G illumination of an all-perovskite tandem are shown in Figure 3a,d (relaxation time constants can be found in Table S4). An LED solar simulator was used to achieve spectral control, where the intensity of 22 different wavelengths in the wavelength range 350–1200 nm can be individually set. To selectively characterize the wide gap subcell, the narrow gap subcell signal was suppressed by using 1 sun intensity illumination in the 750–1200 nm range, which is only absorbed by the narrow gap subcell. The intensity of the 350–550 nm range, which is primarily absorbed by the wide gap subcell (Figure S1), was dialed down from 1 to 0.01 sun intensity to amplify the wide gap subcell signal and study how the EIS signal changes over this intensity range (Figure 3b,e). To study the narrow gap subcell, the intensity of the 350–550 nm range was set to 1 sun, while the 750–1200 nm range was dialed down from 1 to 0.01 sun (Figure 3c,f). The 550–750 nm range was not used here, as it is absorbed by both subcells. Note that sun intensity refers to the intensity the specific wavelength ranges have in the AM1.5G spectrum; see Figure S6 for the irradiance data of all spectra used. We note that because the 550–750 nm range in which both subcells absorb is omitted, the total irradiance of each subcell is lower than for the AM1.5G illumination used for SJs (Figure S4, Table S3), and care should be taken when comparing the results from SJs to subcells directly.

**Figure 3:**
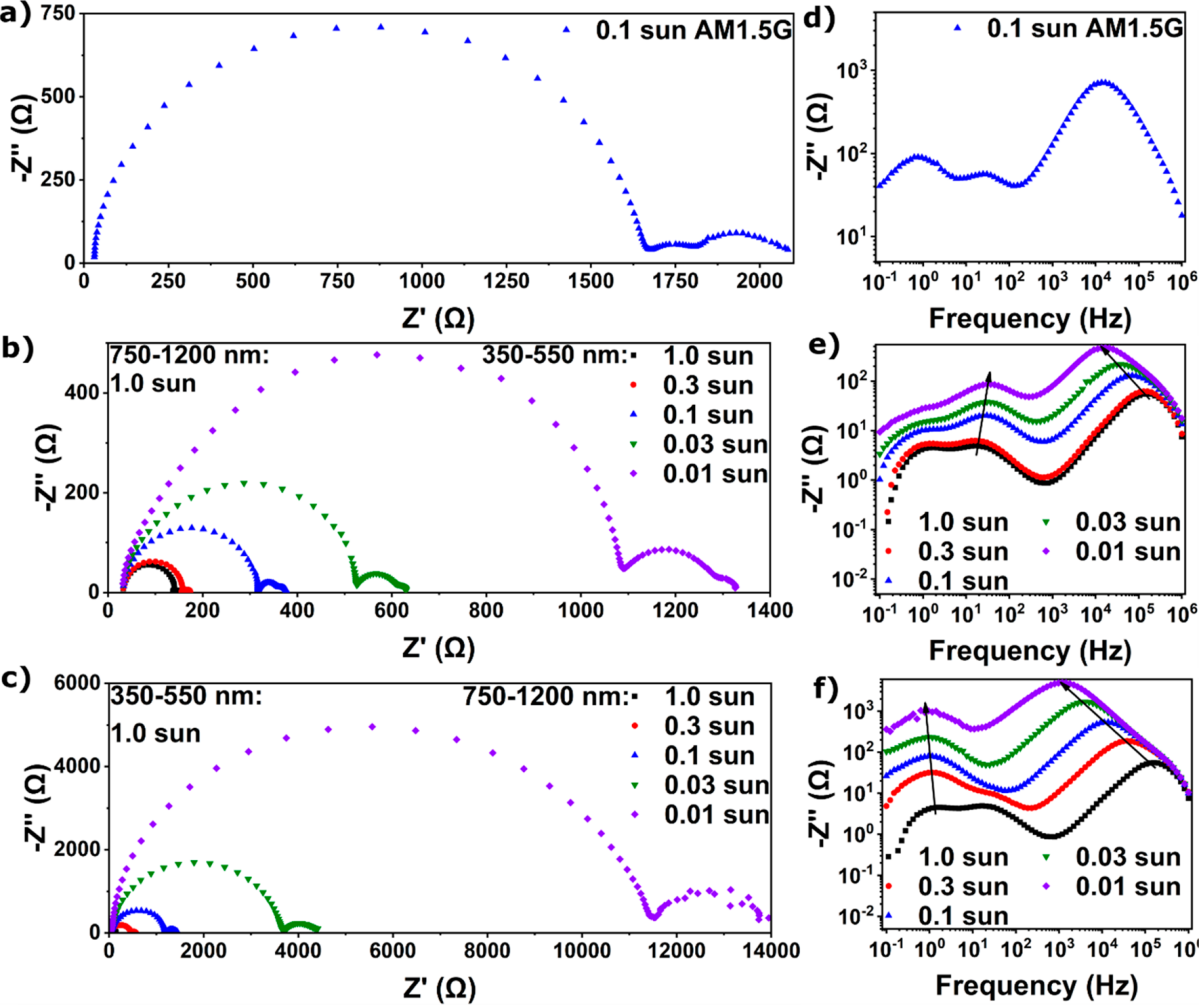
Nyquist plots of an all-perovskite tandem solar cell (a) with 0.1 sun full spectrum AM1.5G illumination, (b) with 1.0 sun 750–1200 nm light and varying 350–550 nm light, allowing the wide bandgap subcell to be studied, and (c) with 1.0 sun 350–550 nm light and varying 750–1200 nm light, allowing the narrow bandgap subcell to be studied. −*Z*″ vs frequency plots of an all-perovskite tandem solar cell (d) with 0.1 sun full spectrum AM1.5G illumination, (e) with 1.0 sun 750–1200 nm light and varying 350–550 nm light, and (f) with 1.0 sun 350–550 nm light and varying 750–1200 nm light. Devices were measured at *V*_*OC*_, with a wait time of 3 min before each measurement to allow *V*_*OC*_ to stabilize. Note the different scales of the *x*-axis in (a–c).

The Nyquist plots in Figure 3b,c illustrate again the large increase in resistance when the intensity of the illumination is reduced from 1.0 to 0.01 sun, with the exception that the contribution of the subcell illuminated at 1.0 sun should stay the same and that the increase in signal is dominated by the signal originating from the subcell of interest. The −*Z*″ vs frequency plots in Figure 3e,f also show that the contribution of the subcell illuminated at 1.0 sun stays constant, whereas the signal of the subcell of interest increases and displays a shift in the apex frequency. It is now possible to extract the relaxation time constants by fitting the Nyquist plots with the SJ EC or by using the apex frequency of the −*Z*″ vs frequency plots (Table S5). Relaxation time constants are on the same order of magnitude as those found for the corresponding SJ devices (Table S3 and Figure S7), validating the selective illumination method. Note, however, that the illumination spectra and intensities used here are different for single junctions and for their tandem subcell counterparts. For a quantitative comparison between single junctions and their tandem subcell counterparts, the incident illumination spectra have to be matched precisely, to ensure carrier densities are the same for both devices. For both EC fitting and apex frequency, full spectrum illumination (Figure 3d) gives accurate ionic relaxation time constants but only one electronic recombination relaxation time constant (which happens to match well with the narrow gap subcell). On the other hand, using selective illumination (Figure 3e,f) gives relaxation time constants that match well between EC fitting and using the apex frequency and SJ devices (Table S3).

Overall, using the selective illumination method yields results that are more consistent with SJ devices and between the EC fitting and apex frequency methods of finding relaxation time constants. Additionally, the approach makes it immediately clear which subcell the extracted relaxation time constants belong to. We emphasize that this information cannot be extracted using full spectrum AM1.5G light and tandem EC.

We now use the selective illumination method to study the effect of passivating the wide gap subcell on electronic recombination and ionic motion in both the wide gap and narrow gap subcells of an all-perovskite tandem solar cell (Figure 4). PDAI_2_ was used as passivating agent, which was found to increase all performance parameters of bromide-rich SJ perovskite solar cells (Table S6).^17^ EIS analysis of SJs (Figure S8, Table S7) shows that passivation increases the electronic recombination resistance ∼1.7 times (from 3240 to 5370 Ω) and the electronic recombination time constant ∼1.5 times (from 42 to 60 μs). The ionic time constant increases ∼1.6 times (from 1.3 to 2.1 ms). These findings are in agreement with reports that passivation reduces the number of mobile defects and nonradiative recombination, leading to improved device performance.^17^ PL mapping of wide gap SJs shows an increase in PL intensity by a factor of ∼5, confirming that PDAI_2_ passivation reduces the density of defect states (Figure S9). For the all-perovskite tandem, using full spectrum AM1.5G illumination for EIS does not yield any useful information (Figure S10). However, the selective illumination method clearly shows that electronic recombination resistance, electronic recombination time constant, and ionic time constant for the wide gap subcell have increased significantly (Figure 4a,c, Table 3, Table S8). The electronic recombination resistance and time constant increase ∼2.0 times (from 2160 to 4290 Ω and from 21 to 43 μs, respectively), and the ionic time constant increases ∼2.2 times (from 2.7 to 6.0 ms), showing that PDAI_2_ passivation effectively improves the performance of wide gap subcells in all-perovskite tandems as well. We showed that time constants decrease with increasing *V*_*OC*_ (Table S3, Figure S5). The *V*_*OC*_ of passivated devices is ∼40 mV higher than the *V*_*OC*_ of control devices, which should decrease the time constants of passivated devices compared with control devices. This makes the fact that the time constants have actually increased even more significant. These results are again supported by a PL intensity increase by a factor of ∼3 for the passivated wide gap subcell, compared to the control wide gap subcell (Figure S10). The electronic recombination resistance and time constant of the narrow gap subcell increase ∼1.1 times (from 3880 to 4300 Ω and from 57 to 64 μs, respectively), and the ionic motion time constant increases ∼1.2 times (from 34 to 41 ms). PDAI_2_ influences the number and time constant of charge carriers transported from the wide gap perovskite to the recombination junction.^17,39^ PDAI_2_ passivation of the wide gap subcell will thus also affect electronic and ionic processes at the interface between the recombination junction and the narrow gap subcell by determining how many charge carriers are available for recombination and ionic processes.^35^ Examples of ionic processes that depend on the availability of electronic charge carriers are the photolysis of PbI_2_^40^ and the (de)doping of Sn-based perovskites,^41^ whereas ion-modulated recombination is an example of a process where the availability of ions influences the electronic recombination lifetime. Increased time constants in the narrow gap subcell indicate that charges take longer to reach the recombination layer, potentially because PDAI_2_ forms a charge extraction barrier in the wide gap subcell; further work beyond the scope of this current work would be needed to explore this further. In addition to tracking improvements of individual subcells in the tandem stack as we have done here, the selective illumination method could also be used as quality control, pinpointing problems in batches that perform below the baseline to a specific subcell.

**Figure 4:**
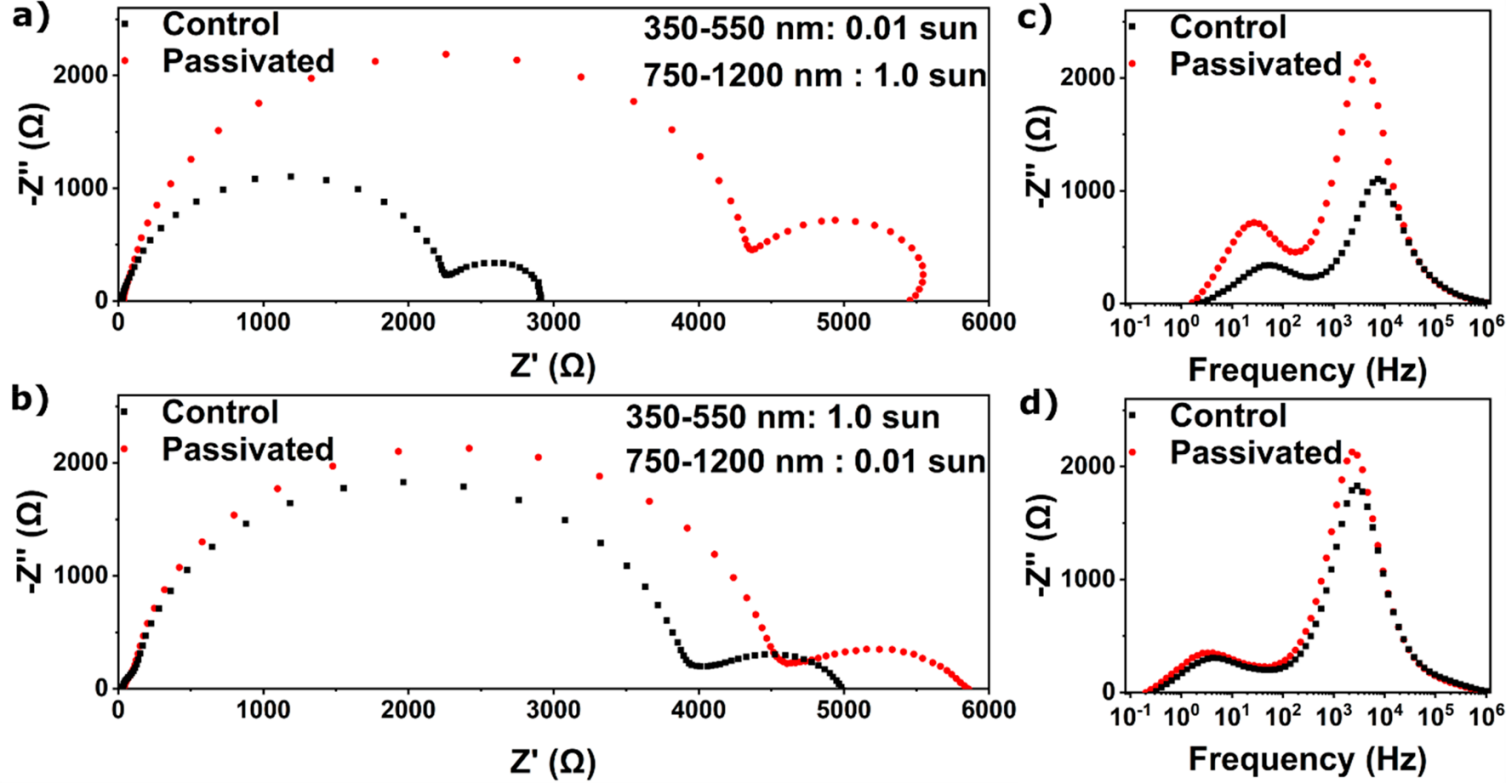
Nyquist plots of a control and a wide gap passivated all-perovskite tandem solar cell (a) with 0.01 sun 350–550 nm light and 1.0 sun 750–1200 nm light, allowing the wide bandgap subcell to be studied, and (b) with 1.0 sun 350–550 nm light and 0.01 sun 750–1200 nm light, allowing the narrow bandgap subcell to be studied. −*Z*″ vs frequency plots of an all-perovskite tandem solar cell (c) with 0.01 sun 350–550 nm light and 1.0 sun 750–1200 nm light and (d) with 1.0 sun 350–550 nm light and 0.01 sun 750–1200 nm light. Devices were measured at *V*_*OC*_, with a wait time of 3 min before each measurement to allow *V*_*OC*_ to stabilize.

**Table 3:** Relaxation Time Constants for Electronic Recombination and Ionic Processes for the Control and Passivated All-Perovskite Tandems in Figure 4^a^

Device	Subcell	Process	R (Ω)	CPE (F)	τ (s)
Control	Wide gap	Recombination	2160	9.73 × 10^–9^	2.1 × 10^–5^
		Ionic	783	3.47 × 10^–6^	2.7 × 10^–3^
	Narrow gap	Recombination	3880	1.48 × 10^–8^	5.7 × 10^–5^
		Ionic	1030	3.28 × 10^–5^	3.4 × 10^–2^
Passivated	Wide gap	Recombination	4290	1.01 × 10^–8^	4.3 × 10^–5^
		Ionic	1290	4.66 × 10^–6^	6.0 × 10^–3^
	Narrow gap	Recombination	4300	1.48 × 10^–8^	6.4 × 10^–5^
		Ionic	1310	3.16 × 10^–5^	4.1 × 10^–2^

a Corresponding resistances, capacitances, and apex frequencies can be found in Table S8.

To explore if EIS can be used to study degradation of all-perovskite tandem solar cells, we subjected an all-perovskite tandem solar cell to 24 h of maximum power point tracking (MPPT) under illumination, during which the tandem degraded to ∼75% of its initial efficiency (Figure 5a). We observe a fast drop in PCE in the first hour of MPPT driven by a decrease in voltage, followed by a slower decay driven by a decrease in current density. The MPPT traces of equivalent wide gap SJ devices only show a small initial decrease in both voltage and current (Figure 5b). Equivalent narrow gap SJ devices show a fast drop in efficiency in the first hour, followed by a slow increase, mainly driven by changes in voltage (Figure 5c). It is important to note that the *V*_*OC*_ of the devices does not decrease under MPPT conditions (Table S10). However, this does not mean that the underlying physical processes that determine *V*_*OC*_ have not changed; in fact, our results indicate that these processes have changed, but their net influence on *V*_*OC*_ is close to zero. The slower decay driven by decreasing current density that was observed for the tandem is absent for both SJ devices. To gain more insight into why the tandem degrades differently than the corresponding individual SJ devices, we performed EIS using selective illumination. Full spectrum illumination clearly shows changes in electronic recombination and ionic time constants but does not provide subcell specific information (Figure S12). Selective illumination allows us to see what is happening in each subcell when the tandem is subjected to MPPT measurements (Figure 5d–g). Surprisingly, the electronic recombination resistance for both subcells has increased significantly following degradation, ∼3.1 times (from 836 to 2580 Ω) for the wide gap subcell and ∼5.0 times (from 560 to 2810 Ω) for the narrow gap subcell. The geometric capacitance on the other hand decreases ∼2.2 times (from 13.4 to 6.06 nF) for the wide gap subcell and ∼2.3 times (from 19.5 to 8.53 nF) for the narrow gap subcell. This results in an increase of the recombination time constant by ∼1.5 times (from 11 to 16 μs) for the wide gap subcell and ∼2.2 times (from 11 to 24 μs) for the narrow gap subcell (Table 4). At the same time, PL mapping shows that PL intensity decreases ∼2 times for the wide gap subcell (Figure S11), whereas the PL intensity increases ∼10 times for the narrow gap subcell (Figure S13). The PL increase for the narrow gap subcell after MPPT is surprising but was found repeatedly in different device batches (Figure S13). Note that Sn-based perovskites need higher illumination intensities for reliable PL measurements (6–10 suns), but due to the relatively short measurement time compared to the 24 h stability test, it is not expected that the PL measurement induces additional changes. Interestingly, EIS shows here that overall nonradiative recombination decreases, while PL shows that radiative recombination decreases for the wide gap subcell but increases for the narrow gap subcell. The decreased geometric capacitance indicates a reduction in surface roughness^42^ or the formation of materials with lower dielectric constants.^24^ These results could potentially be caused by changes in the perovskite absorber materials as well as by the formation of an interfacial charge extraction barrier.

**Figure 5:**
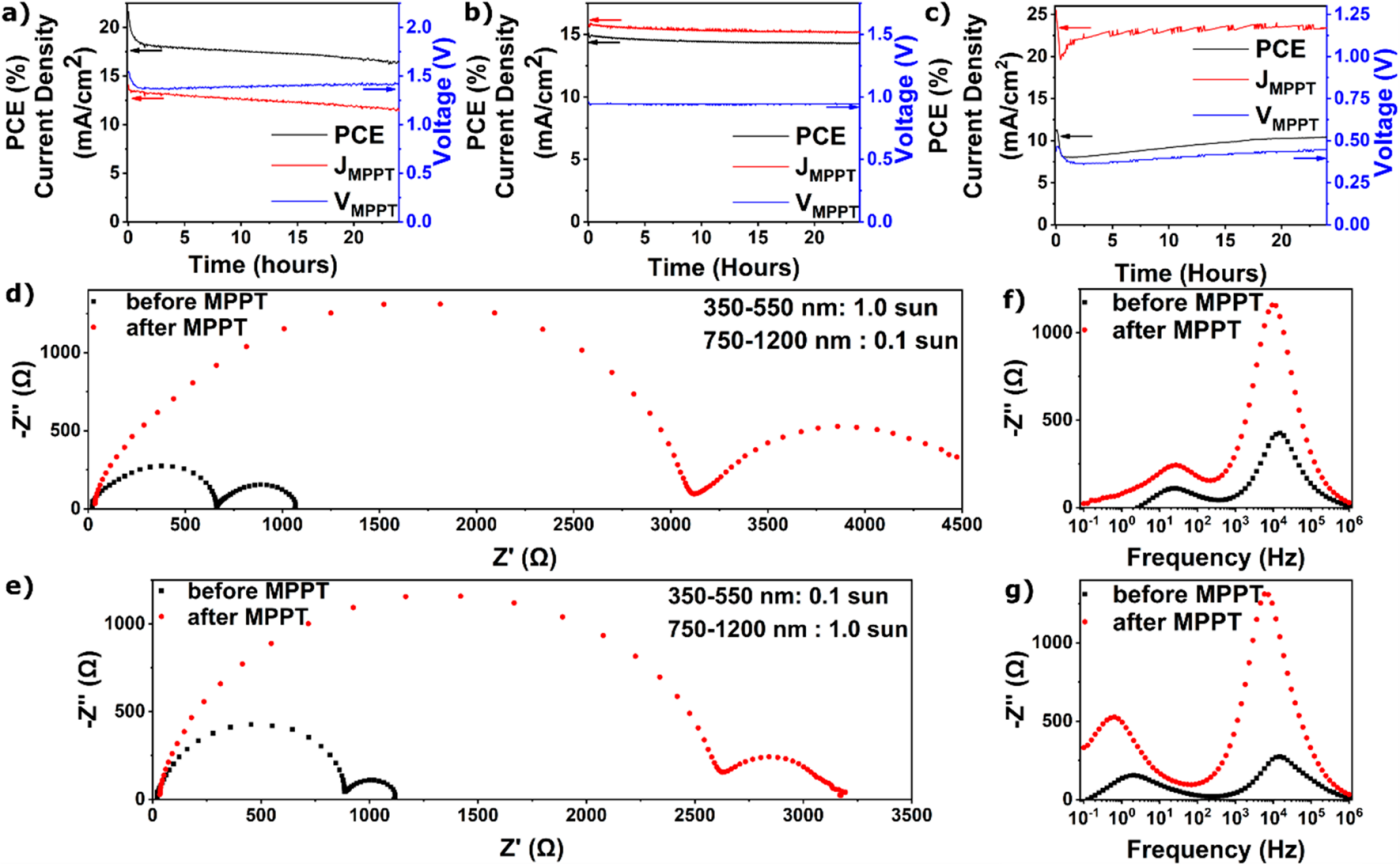
Maximum power point tracking of (a) an all-perovskite tandem solar cell, (b) wide gap SJ, and (c) narrow gap SJ solar cell, showing power conversion efficiency (PCE) during tracking and corresponding current density (*J*_MPPT_) and voltage (*V*_MPPT_). The devices were encapsulated with 25 nm of SnO_*x*_ deposited by atomic layer deposition and measured in ambient air under 1.0 sun intensity AM1.5G illumination. Nyquist plots of an all-perovskite tandem solar cell before and after maximum power tracking for 24 h, (d) with 0.1 sun 350–550 nm light and 1.0 sun 750–1200 nm light, allowing the wide bandgap subcell to be studied, and (e) with 1.0 sun 350–550 nm light and 0.1 sun 750–1200 nm light, allowing the narrow bandgap subcell to be studied. −*Z*″ vs frequency plots of an all-perovskite tandem solar cell (f) with 0.1 sun 350–550 nm and 1.0 sun 750–1200 nm light and (g) with 1.0 sun 350–550 nm light and 0.1 sun 750–1200 nm light. Devices were measured at *V*_*OC*_, with a wait time of 3 min before each measurement to allow *V*_*OC*_ to stabilize.

**Table 4:** Relaxation Time Constants for Electronic Recombination and Ionic Processes before and after MPPT for the All-Perovskite Tandem in Figure 5^a^

Device	Subcell	Process	*R* (Ω)	CPE (F)	τ (s)
Before MPPT	Wide gap	Recombination	836	1.34 × 10^–8^	1.1 × 10^–5^
		Ionic	240	2.49 × 10^–5^	6.0 × 10^–3^
	Narrow gap	Recombination	560	1.95 × 10^–8^	1.1 × 10^–5^
		Ionic	386	2.02 × 10^–4^	7.8 × 10^–2^
After MPPT	Wide gap	Recombination	2580	6.06 × 10^–9^	1.6 × 10^–5^
		Ionic	488	1.21 × 10^–5^	5.9 × 10^–3^
	Narrow gap	Recombination	2810	8.53 × 10^–9^	2.4 × 10^–5^
		Ionic	1450	1.81 × 10^–4^	2.6 × 10^–1^

a Corresponding resistances, capacitances, and apex frequencies can be found in Table S9.

To investigate further, we performed UV/vis absorption measurements (Figure S14) on all-perovskite tandems before and after 24 h of MPPT. Despite an ∼20% drop in current density, there is no appreciable drop in absorption, indicating that the perovskite materials are still absorbing the same amount of light and that charge transport and extraction may be limiting the obtainable current density. EQE (Figure S15) measurements show that for both subcells current density decreases by ∼15% after the MPPT procedure. The observation that current decreases equally for both subcells points toward the recombination layer or the subcell/recombination layer interface as the origin of the decreasing current. The recombination layer electrically connects the two subcells. Its main functions are to provide a solvent barrier to protect the wide gap top cell during processing of subsequent layers and to facilitate efficient recombination between electrons from the wide gap subcell and holes from the narrow gap subcell. In our tandem stack, SnO_*x*_ serves as solvent barrier, and a 1 nm Au layer facilitates recombination (inset Figure 1a). Au is known to migrate under electrical bias,^43^ which could lead to a gradual decline of the recombination facilitating qualities of the recombination layer. Alternatively, a charge extraction barrier could form at the subcell/recombination layer interface. A charge extraction barrier at the Au/HTM interface, for example through the formation of gold-iodide complexes^44^ or electrochemical oxidation of Sn,^45^ could explain the recombination dynamics found by EIS and PL. If an extraction barrier is formed at the narrow gap subcell/recombination layer interface during the MPPT procedure, charges generated in the wide gap subcell can still easily travel to the Au recombination junction, where recombination is limited by the availability of charges from the narrow gap subcell, which have to overcome the newly formed charge extraction barrier before recombining, prolonging the electronic lifetime for charges from the wide gap subcell, without increasing radiative recombination in the wide gap subcell, as the charges in the recombination junction cannot recombine radiatively. Further evidence for this hypothesis is that the electronic and ionic lifetimes of a wide gap single junction device do not significantly change after MPPT (Figure S16 and Table S11), indicating that the observed changes in the tandem stack are likely related to the recombination junction. Meanwhile, in the narrow gap subcell, charges are extracted slower as a result of the charge extraction barrier, leading to reduced quenching of the radiative component, explaining the increased PL. This hypothesis can explain the increased (nonradiative) recombination time constant in both subcells. The observations that the (nonradiative) recombination time constant increases less for the wide gap subcell than for the narrow gap subcell and that radiative recombination (from PL) decreases in the wide gap subcell indicate that some degradation takes place in the wide gap subcell. Further confirmation that a charge extraction barrier is formed in the narrow gap subcell is the 3 times increased ionic time constant for the narrow gap subcell, whereas the ionic time constant for the wide gap subcell remains the same. This indicates that the narrow gap subcell has undergone chemical changes, whereas this is not the case for the wide gap subcell, providing further evidence that an extraction barrier is formed in the narrow gap subcell. We show here that EIS can provide crucial insights into nonradiative recombination and ionic processes. In combination with other characterization techniques, EIS can offer a comprehensive picture of processes happening in perovskite solar cells, help to identify potential bottlenecks, and improve efficiency and stability of these devices,

EIS is a powerful technique to study processes such as electronic recombination and ionic processes in devices under operating conditions. The EIS signal of all-perovskite tandems is much more complex than that of SJ perovskite devices, with overlapping signals complicating the physical interpretation. In many cases, EC modeling does not give satisfactory values for the modeled parameters. Even in the cases where modeling does work, it is not obvious to which subcell each parameter belongs. Illumination intensity can strongly modulate the EIS signal, and it is shown that for both wide gap and narrow gap perovskite SJs the apex frequency significantly shifts, and the impedance can be reduced by several orders of magnitude by increasing illumination intensity. This can be exploited by making use of the absorption properties of the individual subcells in the tandem stack. The wide gap subcell selectively absorbs wavelengths <550 nm; thus, selective illumination (1.0 sun intensity) with 350–550 nm light suppresses the signal from the wide gap subcell and allows the selective measurement of the narrow gap subcell. Similarly, the narrow gap subcell selectively absorbs wavelengths >750 nm. Using this methodology, electronic recombination and ionic relaxation time constants can be extracted and assigned to individual subcells. Using the selective illumination method, we show that PDAI_2_ passivation increases the performance of wide gap subcells, similar to wide gap SJs. In addition, we find that during MPPT a charge transport barrier is formed at the recombination layer/narrow gap subcell interface, indicating that replacing Au in the recombination layer with other conductive materials could improve the stability of all-perovskite tandems. We demonstrate the versatility of the selective illumination EIS method in achieving a better understanding of all-perovskite tandems. In combination with other advanced characterization techniques, such as elemental mapping and transient lifetime measurements, EIS will be a powerful tool to understand the recombination and degradation processes in all-perovskite tandem solar cells. An enhanced understanding of how all-perovskite tandems operate will allow targeted methods to address bottlenecks and ultimately allow these devices to achieve their full potential. The selective illumination method can also be applied to other tandem solar cell technologies, such as silicon-perovskite tandems, to better understand these devices.

## Methods

### Perovskite Solution Preparation

#### Cs_0.25_FA_0.75_Pb(I_0.767_Br_0.233_)

A 0.85 M solution of Cs_0.25_FA_0.75_Pb(I_0.767_Br_0.233_) was prepared by dissolving 0.6375 M formamidinium iodide (FAI, Greatcell Solar), 0.2125 M cesium iodide (CsI, Sigma-Aldrich), 0.3825 M lead bromide (TCI), and 0.476 M lead iodide (PbI_2_, TCI) in a 4:1 (vol:vol) mixture of N,N-dimethylformamide (DMF, Sigma-Aldrich) and dimethyl sulfoxide (DMSO, Sigma-Aldrich). The solution was stirred at 50 °C for 2 h and filtered using a 0.22 μm PTFE membrane before use.

#### Cs_0.15_FA_0.85_Pb_0.5_Sn_0.5_I_3_

A 1.35 M solution of Cs_0.15_FA_0.85_Pb_0.5_Sn_0.5_I_3_ was prepared by dissolving 1.1475 M FAI, 0.2025 M CsI, 0.675 M PbI_2_, 0.675 M SnI_2_, and 0.0675 M SnF_2_ in a 3:1 (vol:vol) mixture of DMF/DMSO. The solution was stirred for 2 h and filtered using a 0.22 μm PTFE membrane before use.

### Wide Gap SJ Fabrication

Patterned ITO glass substrate (KINTEC Company) were cleaned using 15 min of sonication in a 2% Hellmanex III (Sigma-Aldrich) solution, followed by 5 min in DI water, 15 min in acetone, and 15 min in isopropanol. The substrates were dried using a nitrogen stream and subjected to a 15 min UV/ozone treatment before being transferred into a nitrogen-filled glovebox. A 1 mM solution of 2PACz in anhydrous ethanol was spin-coated at 3000 rpm (5 s ramp) for 30 s, followed by annealing for 10 min at 100 °C. After cooling down to room temperature, Cs_0.25_FA_0.75_Pb(I_0.767_Br_0.233_) perovskite was deposited onto the substrates by spin-coating at 2000 rpm for 10 s (2 s ramp) and 6000 rpm for 40 s (4 s ramp). Anhydrous chlorobenzene was dripped onto the spinning substrate 20 s before the end of the program. The substrates were then annealed for 30 min at 100 °C.^14^ A 0.25 mg/mL solution of PDAI_2_ (Sigma-Aldrich) in a 1:1 (vol:vol) mixture of isopropanol and toluene was stirred overnight at 50 °C, filtered using a 0.22 μm PTFE membrane, and subsequently spin-coated at 4000 rpm for 20 s, followed by annealing at 100 °C for 5 min.^17^ The substrates were then transferred to a thermal evaporator for deposition of 20 nm of C60 (Sigma-Aldrich), 7 nm of bathocuproine (Sigma-Aldrich), and 120 nm of Cu.

### Narrow Gap SJ Fabrication

Patterned ITO glass substrate (KINTEC Company) was cleaned using 15 min of sonication in a 2% Hellmanex III (Sigma-Aldrich) solution, followed by 5 min in DI water, 15 min in acetone, and 15 min in isopropanol. The substrates were dried using a nitrogen stream and subjected to a 15 min UV/ozone treatment. A filtered (0.45 μm membrane) 3:1 solution of methanol (Sigma-Aldrich) and PEDOT:PSS (Clevios Heraeus Al 4083) was spin-coated on top of the substrates at 4000 rpm (3.5 s ramp) for 30 s, followed by annealing at 140 °C for 20 min. After the substrates were removed from the hot plate, they were immediately transferred to a nitrogen-filled glovebox. Cs_0.15_FA_0.85_Pb_0.5_Sn_0.5_I_3_ was spin-coated at 5000 rpm (4 s ramp) for 50 s. Anisole was dripped onto the spinning substrate 25 s before the end of the program. The substrate was immediately transferred to a hot plate and annealed at 100 °C for 10 min. A 0.5 mg/mL solution of EDAI_2_ (Sigma-Aldrich) in a 1:1 (vol:vol) mixture of isopropanol and toluene was stirred for overnight at 50 °C, filtered using a 0.22 μm PTFE membrane and subsequently spin-coated at 4000 rpm for 20 s, followed by annealing at 100 °C for 5 min.^46^ After cooling to room temperature, 20 nm of C60, 7 nm of bathocuproine (Sigma-Aldrich), and 120 nm of Cu were deposited by thermal evaporation.

### All-Perovskite Tandem Fabrication

Patterned ITO glass substrate (KINTEC Company) was cleaned using 15 min of sonication in a 2% Hellmanex III (Sigma-Aldrich) solution, followed by 5 min in DI water, 15 min in acetone, and 15 min in isopropanol. The substrates were dried using a nitrogen stream and subjected to a 15 min UV/ozone treatment before being transferred into a nitrogen-filled glovebox. A 1 mM solution of 2PACz in anhydrous ethanol was spin-coated at 3000 rpm (5 s ramp) for 30 s, followed by annealing for 10 min at 100 °C. After cooling down to room temperature, Cs_0.25_FA_0.75_PbI_2.1_Br_0.9_ perovskite was deposited onto the substrates by spin-coating at 2000 rpm for 10 s (2 s ramp) and 6000 rpm for 40 s (4 s ramp). Anhydrous chlorobenzene was dripped onto the spinning substrate 20 s before the end of the program. The substrates were then annealed for 30 min at 100 °C. A 0.25 mg/mL solution of PDAI_2_ (Sigma-Aldrich) in a 1:1 (vol:vol) mixture of isopropanol and toluene was stirred overnight at 50 °C, filtered using a 0.22 μm PTFE membrane, and subsequently spin-coated at 4000 rpm for 20 s, followed by annealing at 100 °C for 5 min.^17^ The substrates were then transferred to a thermal evaporator for deposition of 20 nm of C60 (Sigma-Aldrich). A 25 nm SnO_2_ interlayer was deposited by atomic layer deposition (Picosun). Tetrakis(dimethylamino)tin(IV) (TDMASn, EpiValence) was used as a precursor and H_2_O as reactant. The precursor bubbler was heated to 75 °C and the chamber to 100 °C, and the reactant vessel was kept at room temperature. The pulsing sequence consisted of a 0.6 s pulse of TDMASn, 30 s purge, 0.1 s pulse of H_2_O, and 30 s purge, resulting in a growth rate of 0.1 nm/cycle. Following ALD, 1 nm of Au was deposited by thermal evaporation. The substrates were removed from the glovebox, and a filtered (0.45 μm membrane) 3:1 solution of methanol (Sigma-Aldrich) and PEDOT:PSS (Clevios Heraeus Al 4083) was subsequently spin-coated on top of the substrates at 4000 rpm (3.5 s ramp) for 30 s, followed by annealing at 140 °C for 20 min. After the substrates were removed from the hot plate, they were immediately transferred to a nitrogen-filled glovebox. Cs_0.15_FA_0.85_Pb_0.5_Sn_0.5_I_3_ was spin-coated at 5000 rpm (4 s ramp) for 50 s. Anisole was dripped onto the spinning substrate 25 s before the end of the program. The substrate was immediately transferred to a hot plate and annealed at 100 °C for 10 min. A 0.5 mg/mL solution of ethane-1,2-diammonium iodide (Sigma-Aldrich) in a 1:1 (vol:vol) mixture of isopropanol and toluene was stirred for overnight at 50 °C, filtered using a 0.22 μm PTFE membrane, and subsequently spin-coated at 4000 rpm for 20 s, followed by annealing at 100 °C for 5 min.^46^ After cooling to room temperature, 20 nm of C60, 8 nm of bathocuproine (Sigma-Aldrich) and 120 nm of Cu were deposited by thermal evaporation. For stability tests, 25 nm of SnO_2_ was deposited by ALD on top of C60, followed by 120 nm of Cu.

### Solar Cell Characterization

Current–voltage characteristics were collected by using an Arkeo multichannel platform (Cicci Research) and an LED solar simulator (G2 V Sunbrick Base-UV). An aperture mask with an area of 0.1 cm^2^ was used to define the active area. Devices were scanned at a scan speed of 100 mV/s. EQE and UV/vis absorption were measured using a Bentham PVE300 system in transformer mode. A dual xenon short-arc lamp and a quartz halogen lamp were utilized as the light sources, with a swingaway mirror set to 700 nm. A 10 × 10 mm Si reference cell was used to calibrate the power of the probe beam.

### Electrochemical Impedance Spectroscopy

EIS was performed using a Metrohm PGSTAT302N Autolab. Measurements were performed at *V*_*OC*_, and each device was allowed to equilibrate at *V*_*OC*_ for 3 min before each measurement. Spectra were recorded at a frequency range of 1 MHz to 0.1 Hz, with an integration time of 2 s and a perturbation of 40 mV. Spectra were fitted using Nova 1.12 software, employing the SJ and tandem ECs.

### Photoluminescence Mapping

A Photon etc. IMA system was used to carry out wide-field hyperspectral microscopy measurements. A 405 nm continuous wave laser was used for luminescence excitation. The emitted light from the sample was incident onto a volume Bragg grating, which spectrally split the light onto a temperature-controlled CCD camera operating with an operational wavelength of 400–1000 nm. The camera was maintained at 0 °C by using a thermoelectric cooler. By scanning the angle of the grating relative to the incident light, we obtained the spectral information from each point on the sample was obtained. For wide bandgap perovskite samples, image sets were acquired within the range of 640 to 800 nm, while narrow bandgap perovskite samples were imaged within the 880 to 1000 nm range. The step size for all measurements was set to 2 nm. The absolute number of photons at each point was determined using a two-step process.^47^ Sun’s intensity calculation was performed following previous literature.^47^

## Supporting Information Available

EQE measurements; solar cell performance parameters; impedance spectroscopy spectra, equivalent circuits and time constants; illumination spectra; photoluminescence maps; UV/vis (PDF)
