# Supplementary material for: Electrochemical Impedance Spectroscopy of All-Perovskite Tandem Solar Cells
Source: ACS Energy Lett. 2024 Jan 11;9(2):442–53. doi: 10.1021/acsenergylett.3c02018 (PMC10863385; doi:10.1021/acsenergylett.3c02018)
Supplement: Supplementary file 1 — nz3c02018_si_001.pdf [file nz3c02018_si_001.pdf]

Supporting Information

Electrochemical Impedance Spectroscopy of All-Perovskite Tandem Solar Cells

Bart Roose[1*], Krishanu Dey[2], Melissa R Fitzsimmons,[1] Yu-Hsien Chiang[2], Petra J Cameron[3], Samuel D Stranks[1,2*]

[1] *Department of Chemical Engineering and Biotechnology, University of Cambridge, Philippa Fawcett Drive, Cambridge, CB3 0AS, UK*
[2] *Department of Physics, Cavendish Laboratory, University of Cambridge, 19 JJ Thomson Avenue, Cambridge, CB3 0HE, UK*
[3] *Department of Chemistry, University of Bath, Claverton Down, Bath, BA2 7AY, UK*
E-mail: br340@cam.ac.uk; sds65@cam.ac.uk

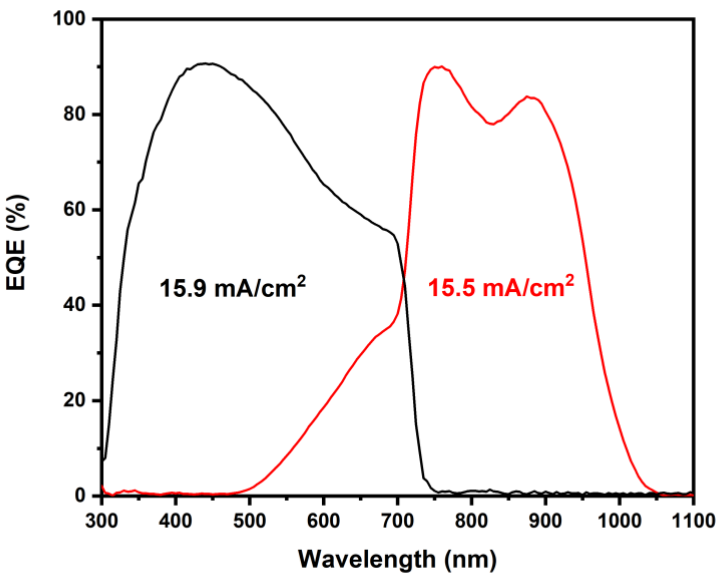

Figure S1
External quantum efficiency (EQE) spectrum of a representative all-perovskite solar cell. EQE spectra of the individual sub-cells are obtained by selectively light biasing the other sub-cell. The short circuit current matches well between *J-V* and EQE measurements.

Table S1
Performance parameters for a batch of wide gap perovskite devices and a batch of narrow gap perovskite devices (8 devices each).

Device	V_{OC} (V)	J_{SC} (mA/cm^2)	FF (%)	Rev PCE (%)	Fwd PCE (%)
Wide gap	1.316 ± 0.007	15.9 ± 0.4	76.4± 1.6	16.0 ± 0.7	16.4 ± 0.6
Narrow gap	0.815 ± 0.018	29.9 ± 0.4	60.7 ± 4.4	14.8 ± 1.3	15.8 ± 1.2

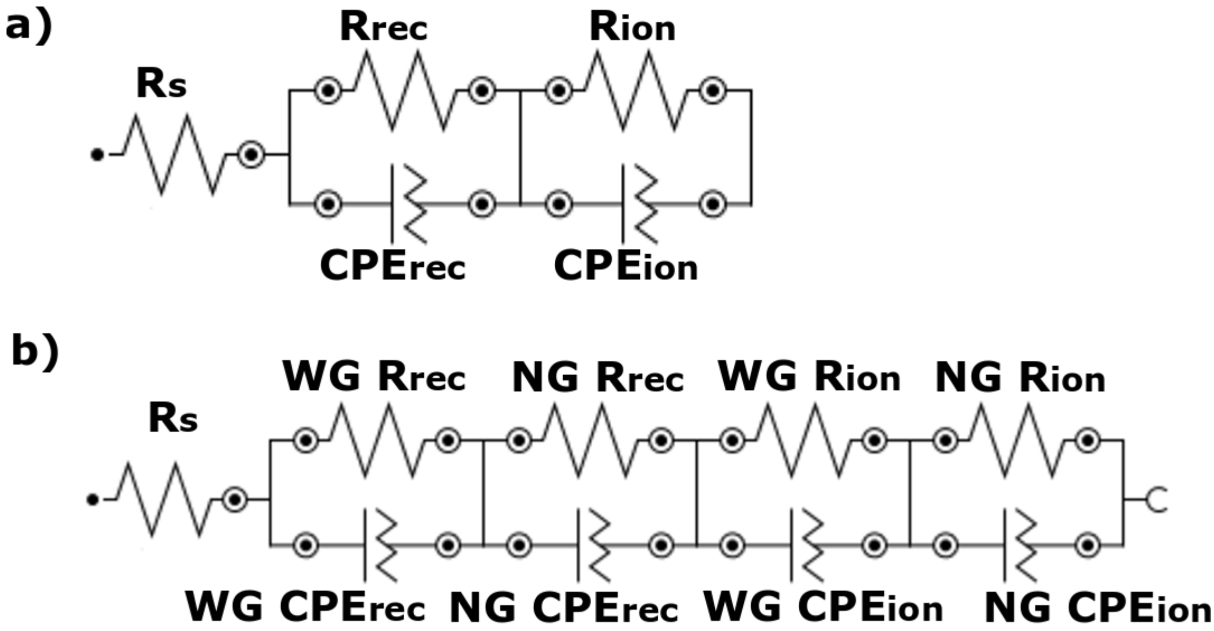

Figure S2
a) SJ EC, where R_s is the series resistance, R_{rec} the recombination resistance, CPE_{rec} the recombination CPE, R_{ion} the ionic resistance and CPE_{ion} the ionic CPE. b) Tandem EC, where WG and NG refer to the circuit elements for the wide gap (WG) and narrow gap (NG) sub-cells, respectively.

Table S2
Resistances, capacitances and apex frequencies for recombination and ionic processes in SJs and tandems in Figure 2, and the corresponding relaxation time constants.

Device	Process	EC fit			Apex frequency	
		R (Ω)	CPE (F)	τ (s)	Frequency (Hz)	τ (s)
Wide gap SJ	Recombination	237	$1.09*10^{-8}$	**$2.6*10^{-6}$**	60600	**$2.6*10^{-6}$**
	Ionic	62.0	$9.29*10^{-4}$	**$5.8*10^{-2}$**	2.63	**$6.1*10^{-2}$**
Narrow gap SJ	Recombination	320	$2.12*10^{-4}$	**$6.8*10^{-6}$**	23800	**$6.7*10^{-6}$**
	Ionic	24.9	$5.40*10^{-3}$	**$1.3*10^{-1}$**	1.03	**$1.5*10^{-1}$**
Tandem	Recombination	629	$9.06*10^{-9}$	**$5.7*10^{-6}$**	25400	**$6.3*10^{-6}$**
	Ionic	19.4	$2.79*10^{-4}$	**$5.4*10^{-3}$**	8.90	**$1.8*10^{-2}$**
		144	$4.70*10^{-3}$	**$6.8*10^{-1}$**	0.340	**$4.7*10^{-1}$**

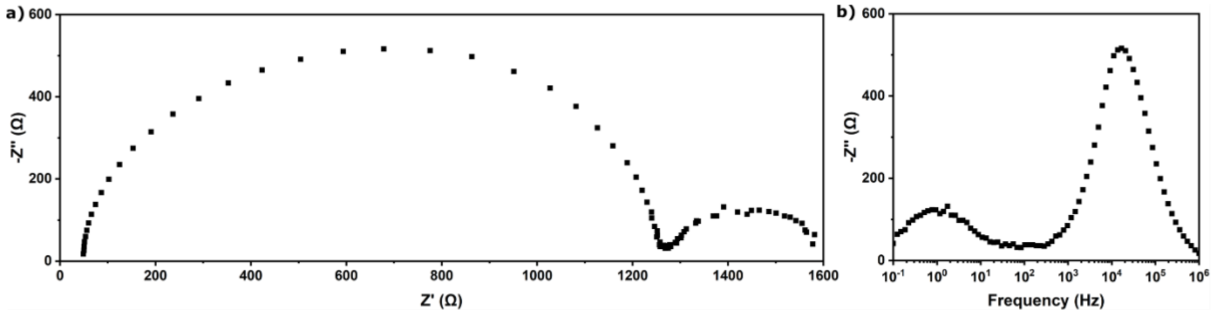

Figure S3

a) Nyquist plot and b) –Z'' *vs* frequency plot of an all-perovskite tandem device, where both the recombination and ionic time constants are very similar for both sub-cells, making it extremely difficult to extract any meaningful information. The device was illuminated at 0.1 sun intensity and measured at V_{oc}, with a wait time of three minutes before each measurement to allow V_{oc} to stabilise.

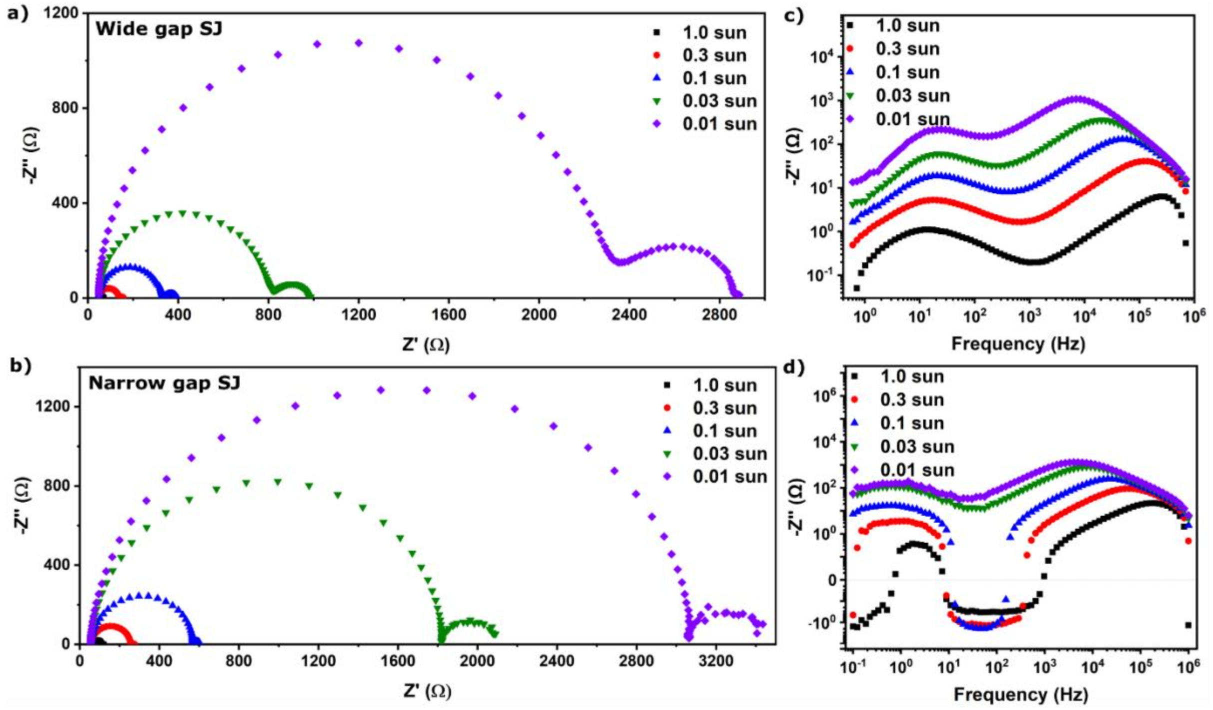

Figure S4

Nyquist plots of a) wide gap SJ and b) narrow gap SJ solar cells. -Z'' vs frequency plots of c) wide gap SJ and d) narrow gap SJ devices. Devices were illuminated at different full spectrum AM1.5G intensities and measured at V_{oc}, with a wait time of three minutes before each measurement to allow V_{oc} to stabilise. The apex frequencies of all semicircle shifts to lower frequencies for both wide and narrow gap SJs.[1] The increased non-radiative recombination time constant is also in line with previous reports on carrier lifetimes.[2] The apex frequency of the low frequency semicircle of the narrow gap SJ device similarly decreases with intensity, which means the ionic time constant increases as light intensity decreases. A potential cause is that the applied voltage decreases with illumination intensity, and provides less driving force for ionic motion.[3] Additionally, illumination has been shown to increase ionic mobility.[4,5] Contrary to this trend of increased time constant with decreased light intensity, is the behaviour of the low frequency semicircle of the wide gap SJ device. A possible explanation is that in mixed-halide perovskites as studied here, I, Br and A-site cations can migrate under illumination, but at different timescales.[5] As light intensity decreases, the slower moving ions contribute disproportionally less to ionic processes, leading to an overall increase in the ionic time constant.

Alternatively, defect density could play a role, as defects have been shown to facilitate ionic motion.[6,7] At low light intensities more defect states remain unfilled, increasing ionic motion. Values for recombination and ionic relaxation time constants, derived both from fitting the Nyquist plots with the SJ EC and from the apex frequency of the -Z'' *vs* frequency plot, can be found in Table S8. Relaxation time constants derived from both methods show consistent results, especially for the lower light intensities. Note that an inductive loop is present for intensities <0.1 sun in the narrow gap Nyquist plot. This phenomenon has been linked to the accumulation of defects at the perovskite/charge extraction layer interface.[8]

Table S3

Apex frequency, resistance, capacitance and relaxation time constants values for recombination and ionic processes derived from the Nyquist and -Z'' *vs* frequency plots in Figure S6. τ_{apex} and τ_{EC} are the relaxation time constants derived from the -Z'' *vs* frequency plot apex frequency and from fitting the SJ EC.

Device	Process	Parameter	Intensity (suns)				
			1.0	0.3	0.1	0.03	0.01
Wide gap SJ	Recombination	Apex (Hz)	242000	119000	49300	20400	7050
		**τ_{apex} (s)**	**$6.6*10^{-7}$**	**$1.3*10^{-6}$**	**$3.2*10^{-6}$**	**$7.8*10^{-6}$**	**$2.3*10^{-5}$**
		R (Ω)	14.2	89.1	285	784	2230
		CEP (F)	$4.33*10^{-8}$	$1.41*10^{-8}$	$1.08*10^{-8}$	$9.97*10^{-9}$	$9.63*10^{-9}$
		**τ_{EC} (s)**	**$6.1*10^{-7}$**	**$1.3*10^{-6}$**	**$3.1*10^{-6}$**	**$7.8*10^{-6}$**	**$2.1*10^{-5}$**
	Ionic	Apex (Hz)	12.1	17.3	20.6	20.6	24.6
		**τ_{apex} (s)**	**$1.3*10^{-2}$**	**$9.2*10^{-3}$**	**$7.7*10^{-3}$**	**$7.7*10^{-3}$**	**$6.5*10^{-3}$**
		R (Ω)	3.21	17.2	59.1	171	582
		CEP (F)	$3.47*10^{-3}$	$4.83*10^{-4}$	$1.23*10^{-4}$	$3.95*10^{-5}$	$1.14*10^{-5}$
		**τ_{EC} (s)**	**$1.1*10^{-2}$**	**$8.3*10^{-3}$**	**$7.3*10^{-3}$**	**$6.8*10^{-3}$**	**$6.7*10^{-3}$**
Narrow gap SJ	Recombination	Apex (Hz)	196000	57500	25400	7470	4970
		**τ_{apex} (s)**	**$8.1*10^{-7}$**	**$2.7*10^{-6}$**	**$6.3*10^{-6}$**	**$2.1*10^{-5}$**	**$3.2*10^{-5}$**
		R (Ω)	49.0	197	513	1770	3070
		CEP (F)	$1.80E*10^{-8}$	$2.45*10^{-8}$	$1.30*10^{-8}$	$1.12*10^{-8}$	$1.09*10^{-8}$
		**τ_{EC} (s)**	**$8.8*10^{-7}$**	**$2.9*10^{-6}$**	**$6.7*10^{-6}$**	**$2.0*10^{-5}$**	**$3.3*10^{-5}$**
	Ionic	Apex (Hz)	1.74	1.16	0.627	0.627	0.769
		**τ_{apex} (s)**	**$9.1*10^{-2}$**	**$1.4*10^{-1}$**	**$2.5*10^{-1}$**	**$2.5*10^{-1}$**	**$2.1*10^{-1}$**
		R (Ω)	2.79	14.5	42.2	297	453
		CEP (F)	$2.74*10^{-2}$	$1.29*10^{-2}$	$7.51*10^{-3}$	$7.90*10^{-4}$	$4.91*10^{-4}$
		**τ_{EC} (s)**	**$7.6*10^{-2}$**	**$1.9*10^{-1}$**	**$3.2*10^{-1}$**	**$2.4*10^{-1}$**	**$2.2*10^{-1}$**

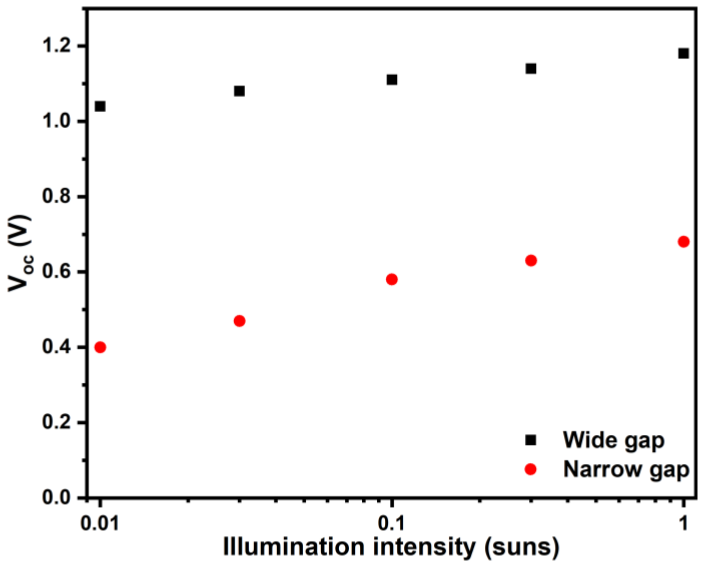

Figure S5

Stabilised open circuit potential (V_{oc}) used for the EIS measurements in Figure 3, as a function of illumination intensity (AM1.5G spectrum) for wide and narrow gap SJ perovskite solar cells. Devices were illuminated for three minutes to let the V_{oc} stabilise before starting the EIS measurement.

Table S4

Resistance, capacitance, apex frequency capacitance and relaxation time constants values for recombination and ionic processes derived from the Nyquist and -Z'' *vs* frequency plots in Figure 3a. Illumination was 0.10 sun AM1.5G.

Device	Process	Equivalent circuit fit			Apex frequency	
		R (Ω)	CPE (F)	τ (s)	Frequency (Hz)	τ (s)
Tandem	Recombination	1650	$6.56*10^{-9}$	$1.08*10^{-5}$	13800	$1.2*10^{-5}$
	Ionic	165	$9.48*10^{-5}$	$1.6*10^{-2}$	24.7	$6.4*10^{-3}$
		240	$1.16*10^{-3}$	$2.8*10^{-1}$	0.769	$2.0*10^{-1}$

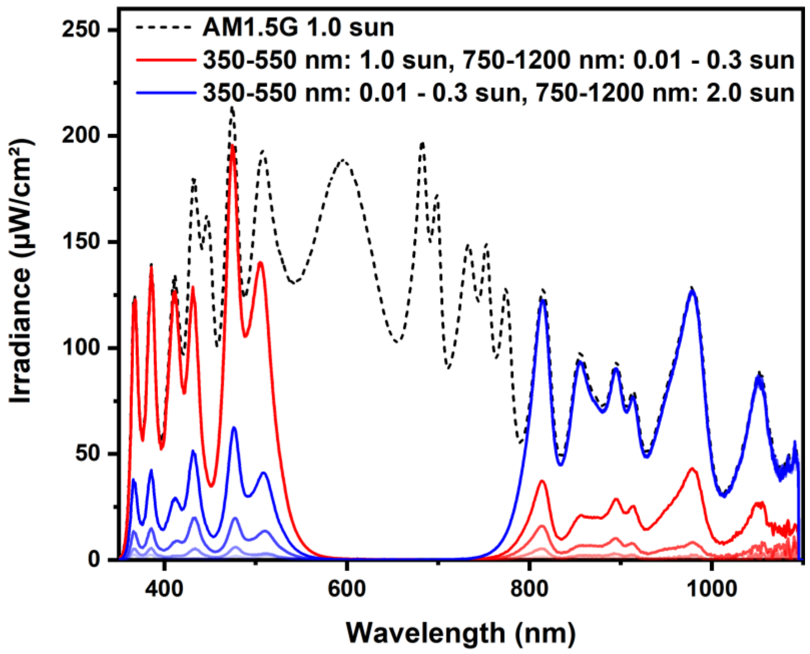

Figure S6

Spectra used for illumination during EIS measurements: full spectrum AM1.5G at 1.0 sun intensity, 350-550 nm at 1.0 sun with decreasing intensity of the 750-1200 nm range (0.3, 0.1, 0.03 and 0.01 sun, red lines), 750-1200 nm at 1.0 sun with decreasing intensity of the 350-550 nm range (0.3, 0.1, 0.03 and 0.01 sun, blue lines).

Table S5

Apex frequency, resistance, capacitance and relaxation time constants values for recombination and ionic processes derived from the Nyquist and –Z'' *vs* frequency plots in Figure 4b,c. τ_{apex} and τ_{EC} are the relaxation time constants derived from the –Z'' *vs* frequency plot apex frequency and from fitting the SJ EC.

Device	Process	Parameter	Intensity (suns)				
			1.0	0.3	0.1	0.03	0.01
Wide gap sub-cell	Recombination	Apex (Hz)	159000	159000	70500	38200	16900
		**τ_{apex} (s)**	**$1.0*10^{-6}$**	**$1.0*10^{-6}$**	**$2.3*10^{-6}$**	**$4.2*10^{-6}$**	**$9.4*10^{-6}$**
		R (Ω)	110	127	287	507	1020
		CEP (F)	$9.25*10^{-9}$	$8.81*10^{-9}$	$7.87*10^{-9}$	$8.03*10^{-9}$	$9.62*10^{-9}$
		**τ_{EC} (s)**	**$1.0*10^{-6}$**	**$1.1*10^{-6}$**	**$2.3*10^{-6}$**	**$4.1*10^{-6}$**	**$9.8*10^{-6}$**
	Ionic	Apex (Hz)	16.4	16.4	30.3	30.3	30.3
		**τ_{apex} (s)**	**$9.7*10^{-3}$**	**$9.7*10^{-3}$**	**$5.3*10^{-3}$**	**$5.3*10^{-3}$**	**$5.3*10^{-3}$**
		R (Ω)	7.26	12.3	47.9	89.1	213
		CEP (F)	$1.61*10^{-3}$	$7.6*10^{-4}$	$1.22*10^{-4}$	$5.74*10^{-5}$	$2.22*10^{-5}$
		**τ_{EC} (s)**	**$1.2*10^{-2}$**	**$9.4*10^{-3}$**	**$5.8*10^{-3}$**	**$5.1*10^{-3}$**	**$4.7*10^{-3}$**
Narrow gap sub-cell	Recombination	Apex (Hz)	159000	38200	13800	4050	1190
		**τ_{apex} (s)**	**$1.0*10^{-6}$**	**$4.2*10^{-6}$**	**$1.2*10^{-5}$**	**$3.9*10^{-5}$**	**$1.3*10^{-4}$**
		R (Ω)	110	423	1120	3540	11400
		CEP (F)	$9.25*10^{-9}$	$9.46*10^{-9}$	$1.12*10^{-8}$	$1.13*10^{-8}$	$1.17*10^{-8}$
		**τ_{EC} (s)**	**$1.0*10^{-6}$**	**$4.0*10^{-6}$**	**$1.3*10^{-5}$**	**$4.0*10^{-5}$**	**$1.3*10^{-4}$**
	Ionic	Apex (Hz)	1.74	1.16	0.943	0.943	0.943
		**τ_{apex} (s)**	**$9.1*10^{-2}$**	**$1.4*10^{-1}$**	**$1.7*10^{-1}$**	**$1.7*10^{-1}$**	**$1.7*10^{-1}$**
		R (Ω)	6.13	86.6	218	711	2940
		CEP (F)	$1.60*10^{-2}$	$1.57*10^{-3}$	$6.91*10^{-4}$	$2.34*10^{-4}$	$5.58*10^{-5}$
		**τ_{EC} (s)**	**$9.8*10^{-2}$**	**$1.4*10^{-1}$**	**$1.5*10^{-1}$**	**$1.7*10^{-1}$**	**$1.6*10^{-1}$**

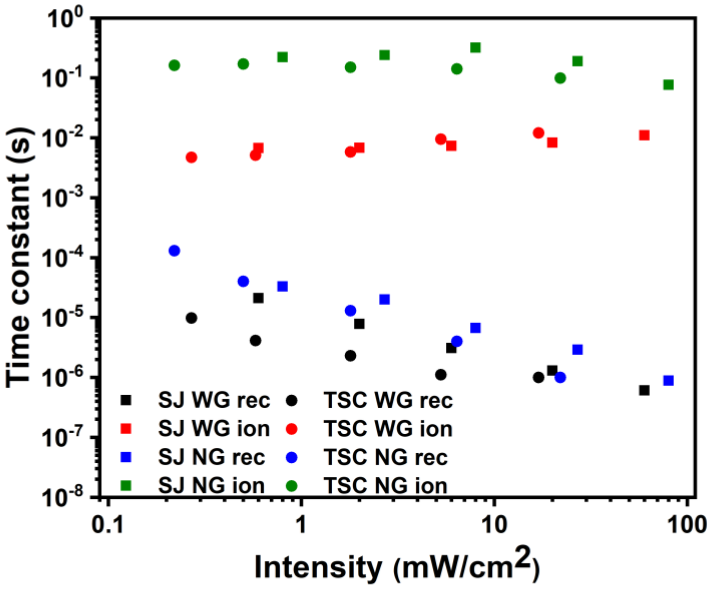

Figure S7

Electronic recombination (rec) and ionic (ion) time constants for single junction (SJ) and tandem subcell (TSC) wide gap (WG) and narrow gap (NG) devices, as a function of the incident illumination intensity. The intensity was recorded using an Avantes Avaspec-ULS2048CL-EVO spectroradiometer.

Table S6

Performance parameters for control and $PDAI_2$ passivated wide band gap perovskite solar cells (18 devices each).

Device	V_{OC} (V)	J_{SC} (mA/cm^2)	FF (%)	Rev PCE (%)	Fwd PCE (%)
Control	1.196 ± 0.003	17.4 ± 0.4	76.7 ± 1.4	15.9 ± 0.4	15.7 ± 0.4
$PDAI_2$ passivated	1.262 ± 0.006	18.1 ± 0.5	78.5 ± 0.9	17.9 ± 0.5	18.2 ± 0.5

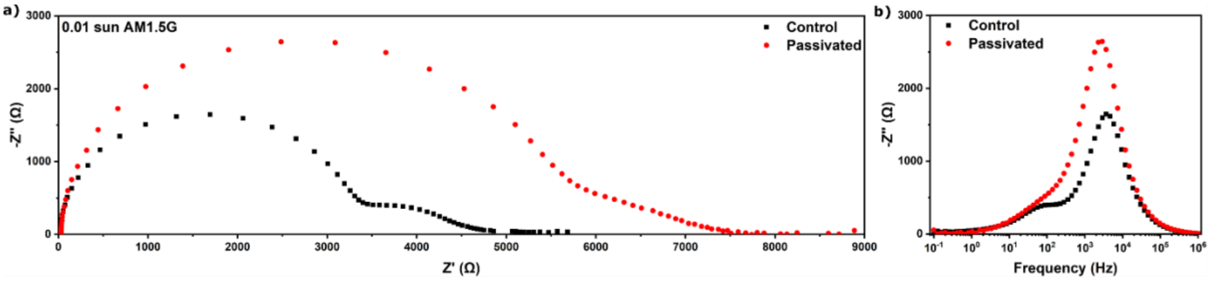

Figure S8

a) Nyquist plot and b) –Z’’ *vs* frequency plot of control and passivated wide gap SJs. The device was illuminated at 0.01 sun intensity and measured at V_{oc}, with a wait time of three minutes before each measurement to allow V_{oc} to stabilise.

Table S7

Apex frequency, resistance, capacitance and relaxation time constants values for recombination and ionic processes derived from the Nyquist and –Z’’ *vs* frequency plots in Figure S8. τ_{apex} and τ_{EC} are the relaxation time constants derived from the –Z’’ *vs* frequency plot apex frequency and from fitting the SJ EC.

Device	Process	EC fit			Apex frequency	
		R (Ω)	CPE (F)	τ (s)	Frequency (Hz)	τ (s)
Control	Recombination	3240	$1.30*10^{-8}$	**$4.2*10^{-5}$**	3680	**$4.3*10^{-5}$**
	Ionic	1950	$6.49*10^{-7}$	**$1.3*10^{-3}$**	111	**$1.4*10^{-3}$**
Passivated	Recombination	5370	$1.11*10^{-8}$	**$6.0*10^{-5}$**	2910	**$5.5*10^{-5}$**
	Ionic	2120	$1.01*10^{-6}$	**$2.1*10^{-3}$**	69.7	**$2.3*10^{-3}$**

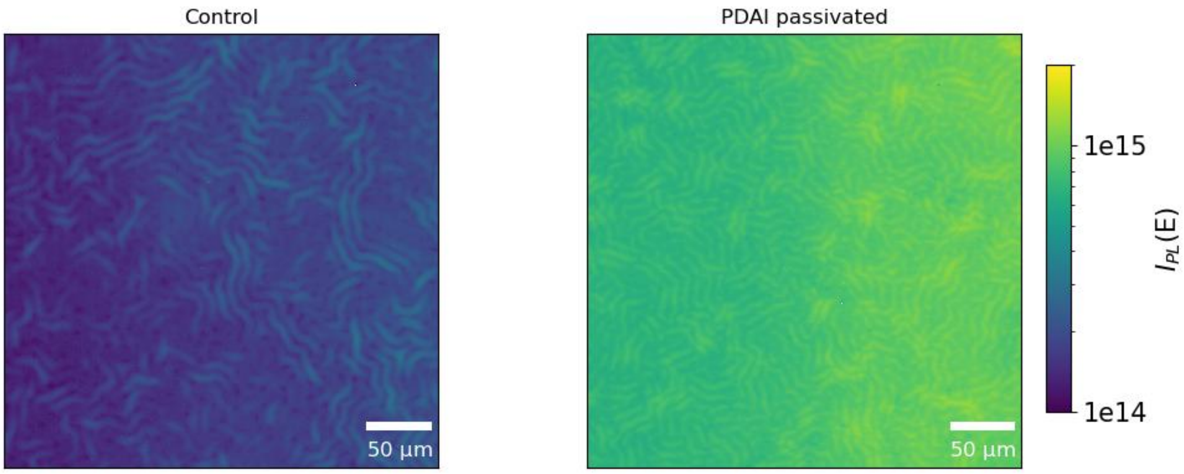

Figure S9

Photoluminescence maps (absolute photon counts) of control and $PDAI_2$ passivated SJ wide gap perovskite solar cells. Excitation intensity was 1 sun equivalent. A 405-nm continuous wave laser was used for excitation, and image sets were acquired within the range of 640 to 800 nm.

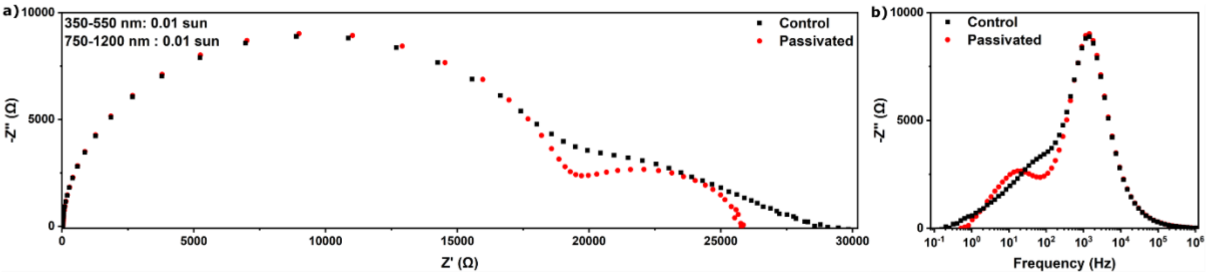

Figure S10

a) Nyquist plot of a control and a wide gap passivated all-perovskite tandem solar cell with 0.01 sun full spectrum AM1.5G illumination, b) -Z'' vs frequency plots of an all-perovskite tandem solar cell with 0.01 sun full spectrum AM1.5G illumination. Devices were measured at V_{oc}, with a wait time of three minutes before each measurement to allow V_{oc} to stabilise.

Table S8

Apex frequency, resistance, capacitance and relaxation time constants values for recombination and ionic processes derived from the Nyquist and –Z'' *vs* frequency plots in Figure 4. τ_{apex} and τ_{EC} are the relaxation time constants derived from the –Z'' *vs* frequency plot apex frequency and from fitting the SJ EC.

Device	Sub-cell	Process	EC fit			Apex frequency	
			R (Ω)	CPE (F)	τ (s)	f (Hz)	τ (s)
Control	Wide gap	Recombination	2160	$9.73*10^{-9}$	**$2.1*10^{-5}$**	7410	**$2.2*10^{-5}$**
		Ionic	783	$3.47*10^{-6}$	**$2.7*10^{-3}$**	54.8	**$2.9*10^{-3}$**
	Narrow gap	Recombination	3880	$1.48*10^{-8}$	**$5.7*10^{-5}$**	2910	**$5.5*10^{-5}$**
		Ionic	1030	$3.28*10^{-5}$	**$3.4*10^{-2}$**	4.20	**$3.8*10^{-2}$**
Passivated	Wide gap	Recombination	4290	$1.01*10^{-8}$	**$4.3*10^{-5}$**	3670	**$4.3*10^{-5}$**
		Ionic	1290	$4.66*10^{-6}$	**$6.0*10^{-3}$**	27.2	**$5.8*10^{-3}$**
	Narrow gap	Recombination	4300	$1.48*10^{-8}$	**$6.4*10^{-5}$**	2300	**$6.9*10^{-5}$**
		Ionic	1310	$3.16*10^{-5}$	**$4.1*10^{-2}$**	4.20	**$3.8*10^{-2}$**

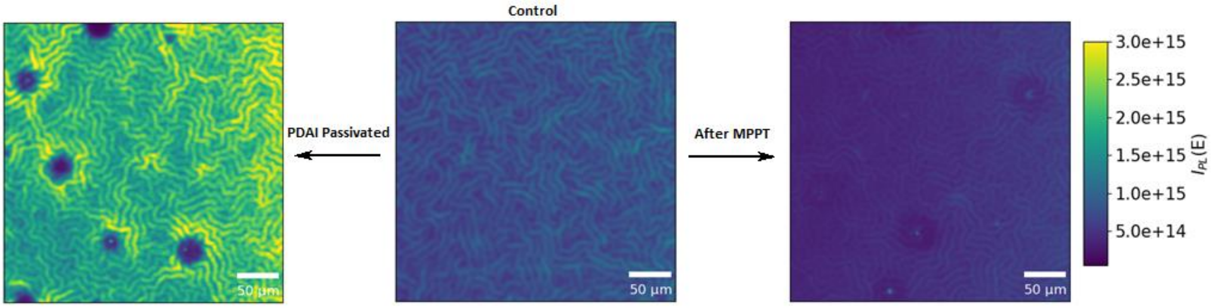

Figure S11

Photoluminescence maps (absolute photon counts) of wide gap sub-cells. The map of the control device is shown in the middle, with $PDAI_2$ passivation on the left, and the control device after 24 hours of MPPT on the right. Excitation intensity was 1 sun equivalent. A 405-nm continuous wave laser was used for excitation, and image sets were acquired within the range of 640 to 800 nm.

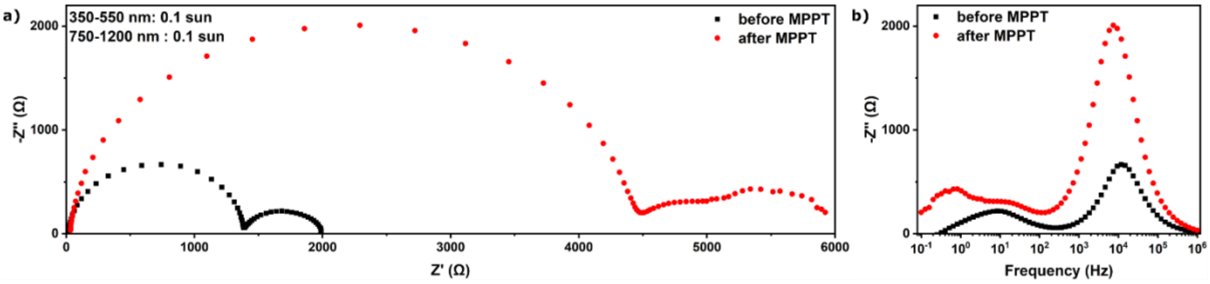

Figure S12

a) Nyquist plot of an all-perovskite tandem solar cell before and after maximum power tracking for 24 hours, measured with 0.1 sun full spectrum AM1.5G illumination, b) -Z'' vs frequency plot with 0.1 sun full spectrum AM1.5G illumination. Devices were measured at V_{oc}, with a wait time of three minutes before each measurement to allow V_{oc} to stabilise.

Table S9

Apex frequency, resistance, capacitance and relaxation time constants values for recombination and ionic processes derived from the Nyquist and –Z'' *vs* frequency plots in Figure 5. τ_{apex} and τ_{EC} are the relaxation time constants derived from the –Z'' *vs* frequency plot apex frequency and from fitting the SJ EC.

Device	Sub-cell	Process	EC fit			Apex frequency	
			R (Ω)	CPE (F)	τ (s)	f (Hz)	τ (s)
Before MPPT	Wide gap	Recombination	836	$1.34*10^{-8}$	$\mathbf{1.1*10^{-5}}$	14900	$\mathbf{1.1*10^{-5}}$
		Ionic	240	$2.49*10^{-5}$	$\mathbf{6.0*10^{-3}}$	27.2	$\mathbf{5.8*10^{-3}}$
	Narrow gap	Recombination	560	$1.95*10^{-8}$	$\mathbf{1.1*10^{-5}}$	14900	$\mathbf{1.1*10^{-5}}$
		Ionic	386	$2.02*10^{-4}$	$\mathbf{7.8*10^{-2}}$	2.08	$\mathbf{7.6*10^{-2}}$
After MPPT	Wide gap	Recombination	2580	$6.06*10^{-9}$	$\mathbf{1.6*10^{-5}}$	9350	$\mathbf{1.7*10^{-5}}$
		Ionic	488	$1.21*10^{-5}$	$\mathbf{5.9*10^{-3}}$	27.2	$\mathbf{5.8*10^{-3}}$
	Narrow gap	Recombination	2810	$8.53*10^{-9}$	$\mathbf{1.6*10^{-5}}$	9354	$\mathbf{1.7*10^{-5}}$
		Ionic	1450	$1.81*10^{-4}$	$\mathbf{2.6*10^{-1}}$	0.648	$\mathbf{2.5*10^{-1}}$

Table S10

Device parameters of tandem and SJ devices, before and after 24 hours of MPPT.

Device	MPPT	V_{OC} (V)	J_{SC} (mA/cm^2)	FF (%)	PCE (%)
Tandem	Before	1.844	14.8	78.8	21.5
	After	1.842	12.9	69.4	16.5
Wide gap	Before	1.254	16.5	76.2	15.8
	After	1.255	16.4	75.9	15.6
Narrow gap	Before	0.779	28.0	61.3	13.4
	After	0.779	27.4	61.8	13.2

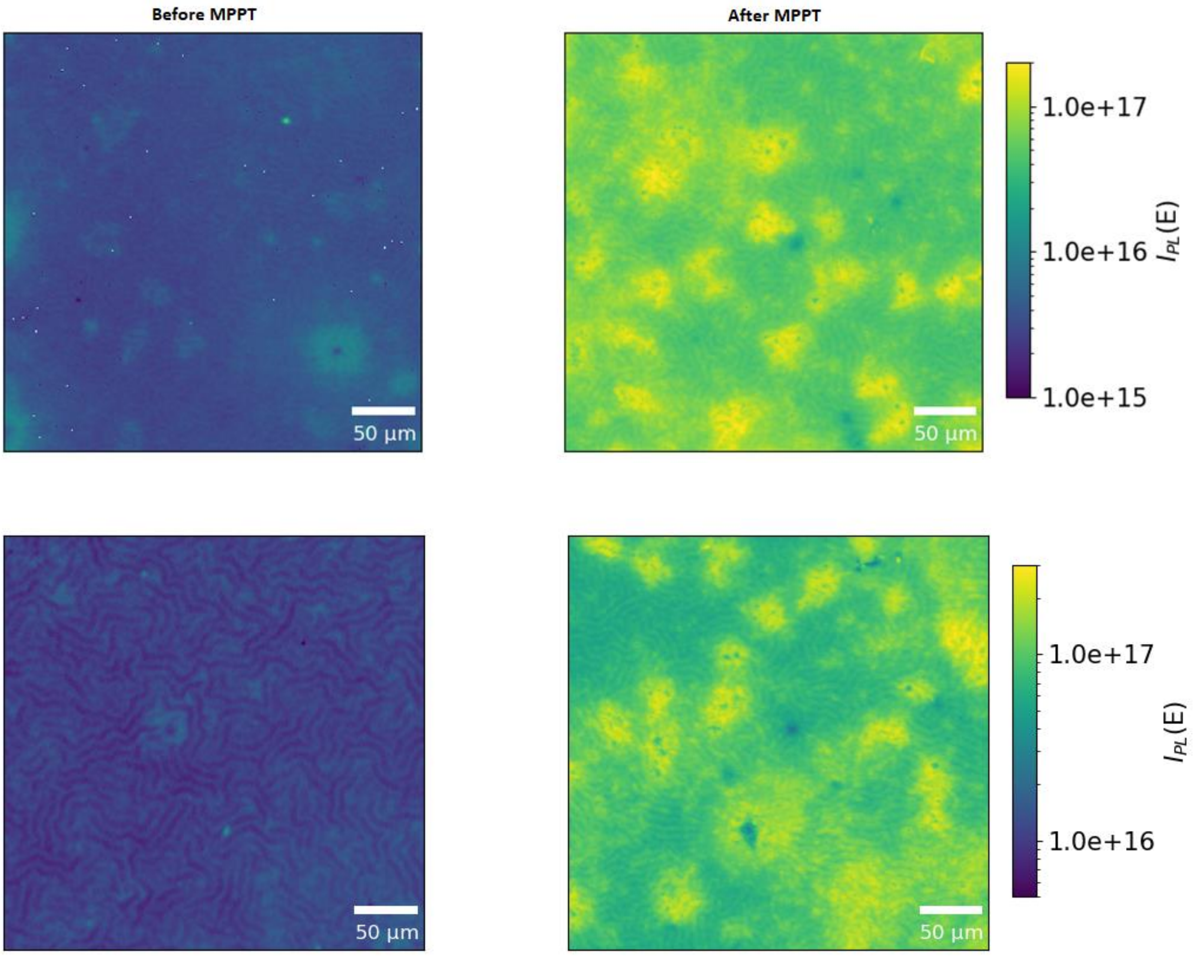

Figure S13

Representative photoluminescence maps (absolute photon counts) of tandem narrow gap sub-cells from different batches, before and after 24 hours of MPPT. Excitation intensity was 6 suns (top) and 10 suns (bottom) equivalent. A 405-nm continuous wave laser was used for excitation, and image sets were acquired within the range of 880 to 1000 nm.

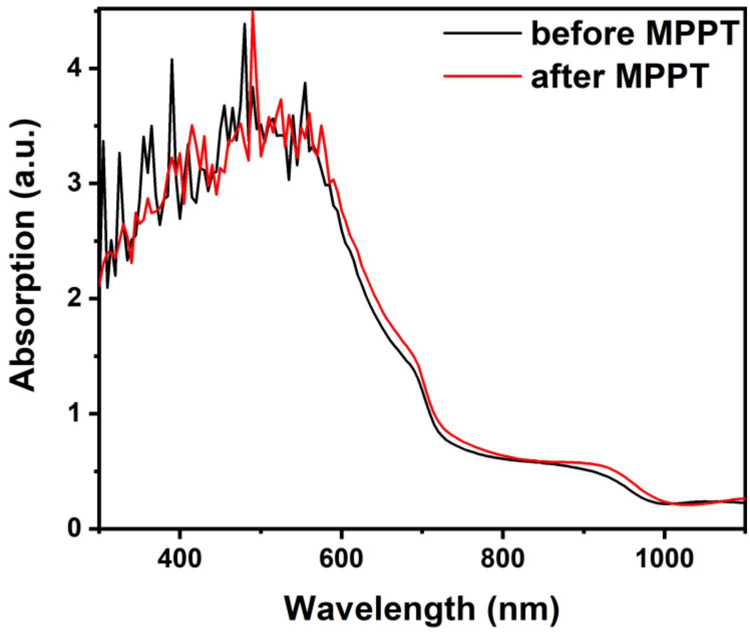

Figure S14
UV/Vis spectra of an all-perovskite tandem solar cell before and after 24 hours of maximum power point tracking.

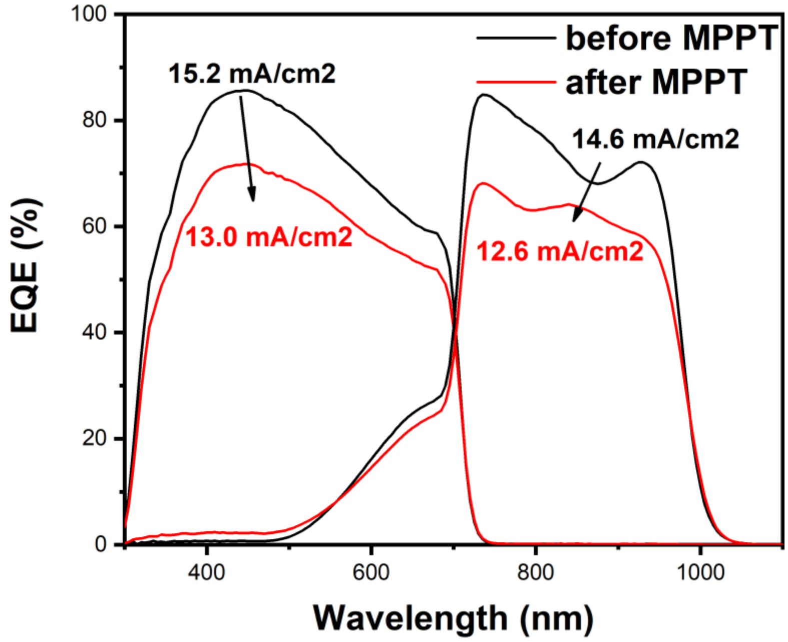

Figure S15
EQE spectra of an all-perovskite tandem solar cell before and after 24 hours of maximum power point tracking.

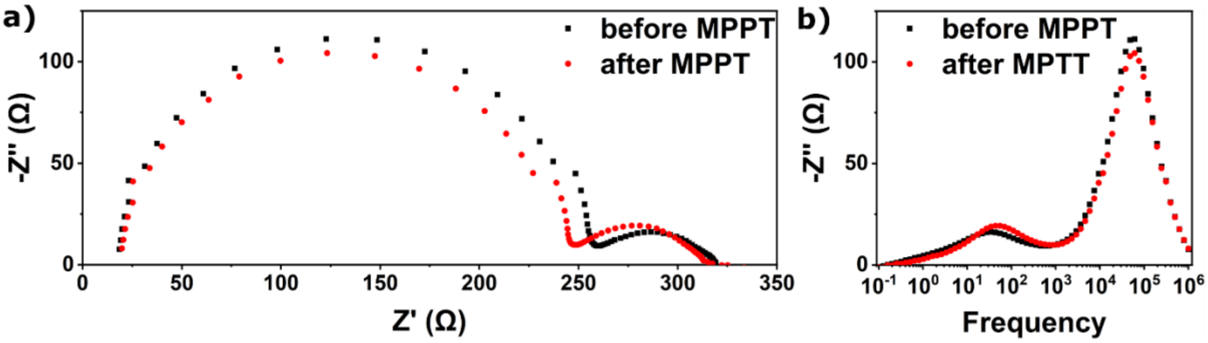

Figure S16
a) Nyquist plot of wide gap single junction perovskite solar cell before and after maximum power tracking for 24 hours, measured with 0.1 sun full spectrum AM1.5G illumination, b) -Z'' vs frequency plot with 0.1 sun full spectrum AM1.5G illumination. Devices were measured at V_{oc}, with a wait time of three minutes before each measurement to allow V_{oc} to stabilise.

Table S11
Apex frequency, resistance, capacitance and relaxation time constants values for recombination and ionic processes derived from the Nyquist and –Z'' *vs* frequency plots in Figure S16. τ_{apex} and τ_{EC} are the relaxation time constants derived from the –Z'' *vs* frequency plot apex frequency and from fitting the SJ EC.

Device	Process	EC fit			Apex frequency	
		R (Ω)	CPE (F)	τ (s)	f (Hz)	τ (s)
Before MPPT	Recombination	242	$1.24*10^{-8}$	**$3.0*10^{-6}$**	60600	**$2.6*10^{-6}$**
	Ionic	62.0	$7.50*10^{-5}$	**$4.7*10^{-3}$**	34.4	**$4.6*10^{-3}$**
After MPPT	Recombination	230	$1.24*10^{-8}$	**$2.9*10^{-6}$**	60600	**$2.6*10^{-6}$**
	Ionic	68.7	$4.42*10^{-5}$	**$3.0*10^{-3}$**	54.8	**$2.9*10^{-3}$**

References

(1) X. Chen; Y. Shirai; M. Yanagida; K. Miyano. Impedance Spectroscopy with Variable Voltages and Illuminations to Reveal Recombination Routes of Free Carriers in Perovskite Solar Cells. In *2019 IEEE 46th Photovoltaic Specialists Conference (PVSC)*; 2019; pp 0645–0647. https://doi.org/10.1109/PVSC40753.2019.8980871.

(2) Wolff, C. M.; Caprioglio, P.; Stolterfoht, M.; Neher, D. Nonradiative Recombination in Perovskite Solar Cells: The Role of Interfaces. *Advanced Materials* **2019**, *31* (52), 1902762. https://doi.org/10.1002/adma.201902762.

(3) Domanski, K.; Roose, B.; Matsui, T.; Saliba, M.; Turren-Cruz, S.-H.; Correa-Baena, J.-P.; Carmona, C. R.; Richardson, G.; Foster, J. M.; De Angelis, F.; Ball, J. M.; Petrozza, A.; Mine, N.; Nazeeruddin, M. K.; Tress, W.; Gratzel, M.; Steiner, U.; Hagfeldt, A.; Abate, A. Migration of Cations Induces Reversible Performance Losses over Day/Night Cycling in Perovskite Solar Cells. *Energy & Environmental Science* **2017**, *10* (2), 604–613. https://doi.org/10.1039/C6EE03352K.

(4) Roose, B. Ion Migration Drives Self-Passivation in Perovskite Solar Cells and Is Enhanced by Light Soaking. *RSC Adv.* **2021**, *11* (20), 12095–12101. https://doi.org/10.1039/D1RA01166A.

(5) McGovern, L.; Grimaldi, G.; Futscher, M. H.; Hutter, E. M.; Muscarella, L. A.; Schmidt, M. C.; Ehrler, B. Reduced Barrier for Ion Migration in Mixed-Halide Perovskites. *ACS Appl. Energy Mater.* **2021**, *4* (12), 13431–13437. https://doi.org/10.1021/acsaem.1c03095.

(6) Li, H.; Zhang, Z.; Jiang, W.; Zhao, C.; Di, H.; Ren, J.; Ou, B.; Xiong, Y.; Liao, F.; Zhao, Y. Low Ion Migration and Defect Density MAPbX3 Single Crystals Grown at Low Temperature for X-Ray

Detection. *J. Mater. Chem. A* **2023**, *11* (30), 16201–16211. https://doi.org/10.1039/D3TA02016A.
(7) Lee, J.-W.; Kim, S.-G.; Yang, J.-M.; Yang, Y.; Park, N.-G. Verification and Mitigation of Ion Migration in Perovskite Solar Cells. *APL Materials* **2019**, *7* (4), 041111. https://doi.org/10.1063/1.5085643.
(8) Khan, M. T.; Huang, P.; Almohammedi, A.; Kazim, S.; Ahmad, S. Mechanistic Origin and Unlocking of Negative Capacitance in Perovskites Solar Cells. *iScience* **2021**, *24* (2), 102024. https://doi.org/10.1016/j.isci.2020.102024.
